# Protein epigenetic scores derived in neonatal saliva as biomarkers of childhood cognition

**DOI:** 10.1038/s41380-026-03702-4

**Published:** 2026-06-24

**Authors:** Rebekah Smikle, Katie Mckinnon, Kadi Vaher, Helen Turner, Hilary Cruickshank, Ray Amir, Yu Wei Chua, Eleanor L. S. Conole, Rebecca M. Reynolds, G. David Batty, Athanasios Tsanas, Lee Murphy, Heather C. Whalley, Riccardo E. Marioni, Simon R. Cox, James P. Boardman

**Affiliations:** 1 Centre for Reproductive Health, Institute for Regeneration and Repair, University of Edinburgh, Edinburgh, UK; 2 Institute for Neuroscience and Cardiovascular Research, University of Edinburgh, Edinburgh, UK; 3 Simpson Centre for Reproductive Health, Royal Infirmary Edinburgh, Edinburgh, UK; 4 Department of Public Health, Policy and Systems, Institute of Population Health, University of Liverpool, Liverpool, UK; 5 Department of Biochemistry, University of Oxford, Oxford, UK; 6 Centre for Cardiovascular Science, Queen’s Medical Research Institute, University of Edinburgh, Edinburgh, UK; 7 Department of Epidemiology and Public Health, University College London, London, UK; 8 Usher Institute, Edinburgh Medical School, University of Edinburgh, Edinburgh, UK; 9 Edinburgh Clinical Research Facility, University of Edinburgh, Western General Hospital, Edinburgh, UK; 10 Institute of Genetics and Cancer, University of Edinburgh, Edinburgh, UK; 11 Lothian Birth Cohorts, Department of Psychology, University of Edinburgh, Edinburgh, UK

**Keywords:** Biomarkers, Neuroscience

## Abstract

Preterm birth is closely associated with immune dysregulation in early life and subsequent learning and psychiatric disorders, but methods for stratifying infants at risk remain elusive. Protein epigenetic Scores (EpiScores) are DNA methylation (DNAm)-based proxies of circulating proteins and can capture health-related exposures such as chronic inflammation. EpiScore of C-reactive protein (DNAm CRP) is associated with inflammatory burden in early life, atypical brain development following preterm birth and adult cognitive ability. To evaluate the utility of neonatal protein EpiScores for predicting childhood cognition, we examined associations of DNAm CRP and 42 other saliva-based EpiScores enriched for inflammatory proteins correlated with low gestational age, with cognition in a cohort of 231 children, including 154 preterm children assessed at 2 years and 127 preterm and term-born children assessed at 5 years. DNAm CRP was negatively associated with 5-year Mullen Scales of Early Learning Composite (ELC) (β = −0.273, *p* = 0.002). Association magnitudes were larger for children born earlier (DNAm CRP x gestational age, β_interaction_ = 0.181). DNAm CD209 was positively associated with 5-year ELC (β = 0.267, adjusted *p* < 0.005). Fourteen other EpiScores were nominally associated with either 2-year Bayley-III Cognitive composite or 5-year ELC (absolute β range 0.180 to 0.245, *p* < 0.05). For preterm children, associations of DNAm CCL18 with 2-year cognition (β = 0.182, *p* = 0.039) and of DNAm CRP (β = −0.318, *p* = 0.021) and DNAm CRTAM (β = −0.307, *p* = 0.008) with 5-year cognition remained significant after adjustment for inflammatory exposures. We demonstrate associations between a range of neonatal salivary EpiScores and childhood cognition, suggesting the clinical value of EpiScores as early life markers of cognitive ability in children at risk of impairment warrants further investigation.

## Introduction

Mounting evidence in the field of developmental programming suggests that perinatal exposure to inflammation increases vulnerability to neurodevelopmental and psychiatric conditions, including autism, cerebral palsy, schizophrenia, depression and even Alzheimer’s disease [1, 2]. Despite the multifactorial and heterogeneous nature of these conditions, immune dysregulation in early life is considered a common pathological component of their aetiology.

Very preterm birth (before 32 weeks’ gestation) is often associated with a characteristic pro-inflammatory profile that arises in the context of multiple morbidities in the perinatal period compared to term infants [3–5]. Elevation of selected pro-inflammatory blood proteins during the first postnatal month is associated with magnetic resonance imaging (MRI) measures of neonatal brain development [4] and cognition in several domains in early childhood [6–12]. However, few studies include a comparator group of term-born controls, and there remains a need to identify early-life markers of immune dysregulation that have functional significance at school entry age, when cognitive demands increase and neuropsychological difficulties emerge [13]. Moreover, measuring selected blood-based proteins at a single time point may not capture baseline and sustained inflammation, as their expression is often phasic [14, 15], and serial blood sampling raises practical and ethical considerations in neonates. Consequently, methods for the early detection of children at risk of cognitive impairment due to immune dysregulation remain elusive.

‘Epigenetic scores’ (EpiScores) based on DNA methylation (DNAm) offer a novel, potentially more stable means of profiling inflammation and other biological processes compared to measuring circulating proteins [16–19]. EpiScores are generated by identifying CpG sites relevant to particular traits, exposures or molecular markers through methods such as epigenome-wide association studies (EWAS) or penalised regression models; weights derived from these analyses are then applied to DNAm in an independent sample to compute EpiScores for that trait, exposure or marker [20]. Protein EpiScores are derived from a linear weighted sum of DNAm at CpG sites that together correlate with protein expression levels [16, 17]. Protein EpiScores often show higher test-retest reliability and stronger associations with disease outcomes than their protein counterparts [18, 19, 21–23]. In previous work we investigated their research utility when derived from neonatal saliva samples because preterm birth is associated with widespread changes in the salivary methylome [24], which we and others have shown is altered in response to inflammation in early life [24–26]. Forty-three saliva-based protein EpiScores enriched for inflammatory proteins were associated with low gestational age at birth in prior work [27], suggesting that they capture both the neonatal physiological response to preterm birth and perinatal inflammation. The proxied proteins associated with preterm birth included immune response proteins, chemokines, growth factors and cell membrane proteins. Notably, of these, DNAm CRP additionally correlates with perinatal inflammatory exposures (histologic chorioamnionitis, sepsis, bronchopulmonary dysplasia and necrotising enterocolitis), and is associated with MRI features of the encephalopathy of prematurity (EoP) [28].

Studies have reported associations between chronic, cumulative inflammation, indexed by protein EpiScores, and neurocognitive outcomes. For instance, multiple EpiScores associate with concurrent cognitive functioning in adult cohorts [18, 21, 29]. Barker et al. [30] show that DNAm CRP in umbilical cord blood is associated with cognition at age 7 years; but to our knowledge, no other protein EpiScores have been investigated in relation to childhood cognitive outcomes. Other studies of protein EpiScores in paediatric populations have primarily focused on MRI features or mental health outcomes, with preterm participants being under-represented despite having differential perinatal DNAm and a high burden of adverse neurobehavioural outcomes [31, 32]. In the context of preterm birth, specific CpG methylation patterns in placenta and buccal tissue have been associated with childhood cognition [33–35]. However, epigenetic proxies of specific proteins, offering a non-invasive measure of perinatal inflammation, may be of translational utility for predicting brain-related health and cognitive outcomes in childhood.

In this study, we linked neonatal saliva-based protein EpiScores, which are associated with gestational age at birth and predominantly reflect immune proteins, with measures of development and cognition at 2 and 5 years of age acquired from a birth cohort enriched for prematurity [36] to test four hypotheses. First, we tested a specifically directional hypothesis focusing on DNAm CRP given the comparatively rich prior literature for this marker: we hypothesised that higher DNAm CRP would be associated with poorer general cognitive ability at 2 and 5 years of age. Second, we investigated whether any of the 42 other EpiScores that are altered in preterm infants are associated with general cognitive ability at 2 and 5 years of age. Third, we examined whether there is an interaction between DNAm CRP and gestational age at birth, hypothesising that the association between DNAm CRP and general cognitive ability would be stronger for children born at lower gestational ages. Fourth, we tested the hypothesis that in preterm children, associations between EpiScores and general cognitive ability are attenuated by accounting for perinatal inflammatory exposures.

## Materials and methods

### Participants

Participants were 2- and 5-year-old children born at < 33 weeks’ gestation and a comparator group of children born at term. All participants were recruited at birth into the Theirworld Edinburgh Birth Cohort (TEBC), a prospective, longitudinal study designed to investigate the impact of preterm birth on brain development and outcomes [36]. Cohort exclusion criteria were major congenital malformations, chromosomal abnormalities, congenital infection, parenchymal brain injury (cystic periventricular leukomalacia, haemorrhagic parenchymal infarction or post-haemorrhagic ventricular dilation). As such, the cohort is representative of the majority of survivors of modern neonatal intensive care practices [36, 37]. Ethics approval was obtained from the National Research Ethics Service (South East Scotland Research Ethics Committee 11/55/0061, 13/SS/0143 and 16/SS/0154). Written informed parental consent was obtained for each participant. Neonatal DNAm data and cognitive assessment data at one or both time points (2 and 5 years) were available for 231 children, which is reflected in the flowchart of the study sample (Supplementary Fig. 1).

### Clinical and demographic measures

Clinical and demographic data were collected through questionnaires administered to the primary caregiver and review of medical records. We included the following perinatal inflammatory comorbidities following definitions from previous work [4]: histologic chorioamnionitis (HCA) was defined as the presence of inflammatory response in the placental membranes of any grade or stage; sepsis was defined as blood culture positive for pathogenic organism (culture positive) or clinically suspected infection treated with intravenous antibiotics for ≥ 5 days (culture negative); necrotising enterocolitis (NEC) was defined as medical treatment for ≥ 7 days or surgical treatment; bronchopulmonary dysplasia (BPD) was defined as the need for supplemental oxygen therapy or respiratory support at 36 weeks corrected gestational age. All comorbidities were coded as binary variables (present/absent). Birthweight z-scores were calculated according to INTERGROWTH-21st standards [38]. Socioeconomic status was based on the Scottish Index of Multiple Deprivation 2016 (SIMD) and maternal education attainment (highest achieved) around the time of birth. SIMD, a neighbourhood-level indicator of socioeconomic circumstances, is derived from a family’s postal code. It uses multiple dimensions to assign a level of deprivation to the neighbourhood, ranked from most deprived (rank 1) to least deprived (rank 6976) [39]. Maternal education, an individual-level indicator of socioeconomic status, was coded as a binary variable of a university undergraduate degree or higher (Yes/No). Maternal cigarette smoking during pregnancy (self-reported) was coded as current versus never/previous.

### DNA methylation and EpiScore calculation

Saliva samples for DNAm were obtained from preterm infants at term-equivalent age and from term infants shortly after birth. Saliva was collected in Oragene OG-575 Assisted Collection kits, by DNA Genotek, and DNA extracted using prepIT.L2P reagent (DNA Genotek, ON, Canada).

Details of methylation measurement have been described previously [27, 28]. Briefly, DNA was bisulphite converted and methylation levels were measured using Illumina HumanMethylationEPIC BeadChip (Illumina, San Diego, CA, USA) at the Edinburgh Clinical Research Facility (Edinburgh, UK). The arrays were imaged on the Illumina iScan or HiScan platform, and genotypes called automatically using GenomeStudio Analysis software 2011.1 (Illumina). DNAm was processed in four batches.

Details of DNAm data pre-processing have also been outlined previously [24, 27]. Raw intensity (.idat) files were read into the R environment (version 3.4.4) using *minfi. wateRmelon* and *minfi* were used for preprocessing, quality control, and normalisation [40, 41]. Specifically, the *pfilter* function in *wateRmelon* was used to exclude samples with 1% of sites with a detection *p*-value > 0.05, sites with beadcount < 3 in 5% of samples, and sites with 1% of samples with detection *p*-value > 0.05. Cross-hybridising probes, probes targeting single nucleotide polymorphisms with overall minor allele frequency ≥ 0.05 and control probes were also removed [42]. Samples were removed if there was a mismatch between predicted sex (*minfi*) and observed sex (*n* = 3), or if samples did not meet preprocessing quality control criteria (*n* = 29). Data were danet normalised which includes background correction and dye bias correction [40]. Saliva contains different cells types, including buccal epithelial cells. Epithelial cell proportions were estimated with epigenetic dissection of intra-sample heterogeneity with the reduced partial correlation method implemented in the R package EpiDISH [43]. Probes located on sex chromosomes were removed before analysis.

The 43 protein EpiScores analysed in this study were previously shown to correlate with low gestational age at birth [27]. Mckinnon et al. calculated these EpiScores using summary statistics from prior studies, where the correlation with proteins was r > 0.1, *p* < 0.05 in independent test sets [16, 17, 27, 30, 44]. The original training datasets for all protein EpiScores except DNAm CRP were the German population-based KORA study (*n* = 944, mean age 59 years) and the Scottish Lothian Birth Cohort 1936 (LBC1936; *n* = 975, mean age 70–73 years). DNAm was quantified in blood using the HumanMethylation450K BeadChip (KORA) and the Illumina 450 K methylation array (LBC1936). Independent validation datasets included a subset of Generation Scotland:STRADL (*n* = 778, mean age 66 years) and the LBC1921 (*n* = 162, mean age 87 years). The CRP EpiScore weights were derived from Barker et al.’s seven-CpG variation of Ligthart et al.’s CRP EpiScore [17, 30], which correlates with perinatal inflammatory exposures and neonatal brain development [28]. Ligthart et al.’s EWAS training dataset included 9 European adult cohorts (*n* = 8863, mean age 60–87 years) and a validation dataset of 4 African American cohorts (*n* = 4111, mean age 41–73 years), using the HumanMethylation450K BeadChip for DNAm quantification in whole blood. In the original studies, protein levels were compared with genome-wide DNAm patterns using EWAS or penalised regression, identifying CpG sites predictive of protein abundance. Each CpG was assigned a weight corresponding to the strength of its association with the protein. In the current study, for each participant, EpiScores were obtained by multiplying the methylation proportion at a given CpG by the effect size from previous studies, as previously described [16, 27].

Principal component analysis of all 43 EpiScores showed evidence of DNAm processing batch effects (Supplementary Fig. 2); therefore, batch was included as a covariate in downstream analyses.

### Cognitive assessments

#### Bayley scales of infant and toddler development

At around 24 months corrected age, preterm children born < 1500 g or < 32 weeks of gestation were assessed using the Bayley Scales of Infant and Toddler Development, Third Edition (Bayley-III) [45] by trained practitioners blinded to EpiScores. The Bayley-III cognitive composite provides a measure of general cognitive ability across memory, information processing, concept formation, problem solving and categorisation; it is an age-standardised index score, with normative mean of 100 and standard deviation (SD) of 15. One hundred and fifty-four participants with neonatal EpiScore data had cognitive data at the 2-year time-point. Their data corresponded to DNAm processing batches 1–4: batch 1 included 74 participants (48.1%); batch 2 included 40 (26.0%); batch 3 included 21 (13.6%); batch 4 included 19 (12.3%).

#### Mullen scales of early learning

At around 5 years of age, children were assessed using the Mullen Scales of Early Learning (MSEL) [46]. The MSEL Early Learning Composite (ELC) provides a measure of general cognitive performance, derived from four subscales: visual reception, fine motor, receptive language and expressive language. ELC is a standard score with normative mean of 100 and SD of 15. In exploratory analyses defined below, we also derived the age-adjusted subscale scores, which are each T*-*scores with mean of 50 and SD of 10. One hundred and twenty-seven participants with neonatal EpiScore data had MSEL ELC data at 5 years of age. Their data corresponded to DNAm processing batches 1 and 2: batch 1 included 50 participants (39.4%); batch 2 included 77 (60.6%).

### Statistical analysis

The analysis plan for this project was preregistered (https://osf.io/pghw2) and all statistical analyses were performed in R (version 4.2.3).

Maternal and infant clinical and demographic characteristics were compared between preterm and term children. Standardised mean difference (Cohen’s d) was used to quantify effect sizes: 0.2 (small), 0.5 (medium), and 0.8 (large) [47]. For continuous variables, we used Mann-Whitney *U*-test to compare distributions. For categorical variables, we used Chi-square tests to compare proportions.

All regression analyses in this study included baseline and adjusted models. Outcomes of interest were Bayley-III Cognitive Composite scores or MSEL Early ELC scores. Covariates for each model are specified below. Covariates were selected based on their potential to act as confounders, variables that influence both EpiScores and the outcome, or because their inclusion could improve the precision of the model estimates. Adjusted models accounted for prematurity-related and socioeconomic variables. Continuous variables were scaled to have a mean of zero and variance of one. In the event of missing data for any covariates, complete case analyses were performed for comparison between baseline and adjusted multivariable models.

Baseline models: outcome of interest ~ EpiScore + gestational age at sampling + sex + batch Adjusted models: outcome of interest ~ EpiScore + gestational age at sampling + sex + batch + gestational age at birth + birthweight Z-score + maternal education + SIMD.

#### DNAm CRP and cognitive ability associations

To determine the effect of neonatal DNAm CRP on Bayley-III Cognitive composite at 2 years or MSEL ELC at 5 years, data were analysed in univariable, baseline and adjusted regression models. Visual inspection of diagnostic plots suggested no regression assumptions were violated (Supplementary Fig. 3). Analyses were performed for whole group, and also stratified by preterm or term children. Given that an interaction effect has been observed between DNAm CRP and gestational age at birth on MRI metrics, such that there were significant relationships found in preterm but not term neonates [28], we repeated analyses to include a product interaction term (DNAm CRP x gestational age at birth), for the whole group and for the preterm subgroup.

In models where DNAm CRP was the predictor variable, we conducted one-tailed significance tests given the specific direction of our preregistered hypothesis, and thus applied a *p*-value significance threshold of < 0.10, reflecting that only an association in the hypothesised direction was considered theoretically meaningful. This preregistered threshold was applied to DNAm CRP because of its known associations with GA, perinatal inflammatory burden and MRI features of EoP in this cohort [27, 28], as well as extensive work in other samples indicating a negative association with neurocognitive outcomes [16, 21, 29, 30, 32, 48].

#### DNAm CRP and MSEL subscale associations

In exploratory analyses, we performed linear regressions to examine associations between DNAm CRP and MSEL subscales in univariable, baseline and adjusted models. Here, we corrected *p*-values for multiple comparisons using the false discovery rate (FDR) method (Benjamini-Hochberg procedure [49]), at the level of MSEL subscales in each model type separately. Significance was deemed at a threshold of p_FDR_ (*q-*value) < 0.05. We further compared the magnitude of associations across domains and determined the relative effects of DNAm CRP on specific cognitive domains. Differences between association magnitudes were assessed using the Williams (1959) [50] test for dependent groups with overlapping correlations, using the “cocor” R package (http://cran.r-project.org/web/packages/cocor/cocor.pdf).

#### Other EpiScore and cognitive ability associations

**Fig. 1:**
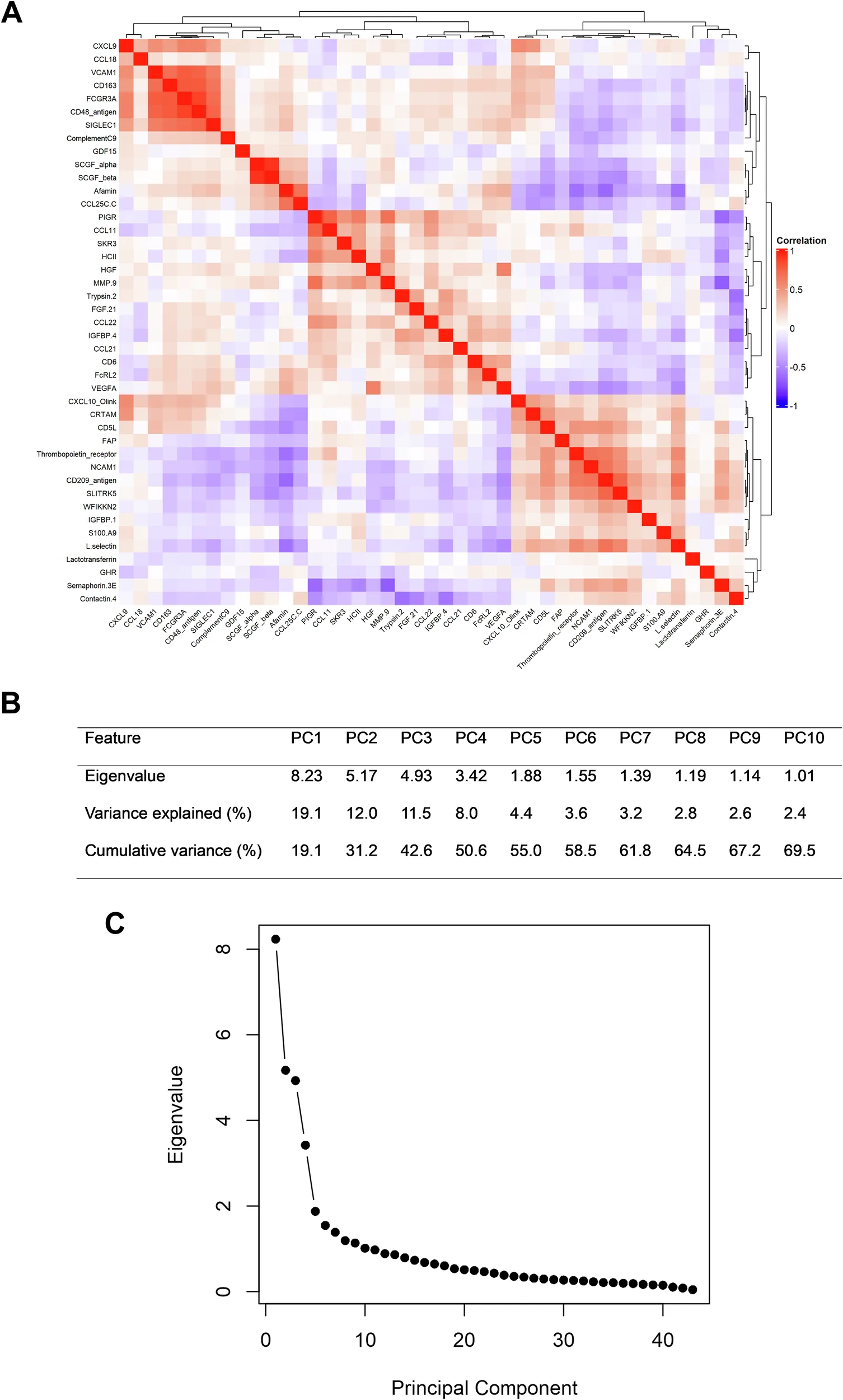
Dimensionality reduction of EpiScores to determine statistical families for correction of multiple comparisons. **A** Heatmap and dendrogram of correlations of EpiScores. Direction and magnitude of Spearman’s correlation coefficients are shown by the colour scale, with red as positive and blue as negative when statistically significant (*p* < 0.05). **B** Principal components analysis eigenvalues and variance explained by each principal component. **C** Scree plot of eigenvalues against principal components of EpiScores. PC principal component.

To determine the associations of the other 42 neonatal EpiScores with Bayley-III Cognitive composite at 2 years or MSEL ELC at 5 years, data were similarly analysed using univariable, baseline and adjusted regression models. Analyses were performed for whole group and preterm subgroup. To correct for multiple comparisons, we first estimated the effective number of independent statistical tests by conducting a principal component analysis (PCA) on the correlation matrix of the EpiScores and applied a PCA-based modification of Bonferroni correction, as described and validated by Gao et al [51, 52]. This approach accounts for the high intercorrelation among EpiScores. The resulting estimate of independent tests allowed us to apply a correction for multiple comparisons that was appropriately stringent without being overly conservative. Spearman’s correlation analysis of EpiScores showed (absolute) correlation coefficients of 4.71 × 10^−5^ to 0.927 (Fig. 1A). Using Kaiser Criterion, ten principal components with eigenvalues > 1 were identified from the correlation of the 42 EpiScores (Fig. 1B; Supplementary Table 1), explaining 69.5% cumulative variance. Accordingly, we calculated the adjusted point-wise significance threshold for individual EpiScores using the Bonferroni formula as 0.005 (<0.05/10). We also report associations which are significant at a nominal threshold (uncorrected *p* < 0.05). Finally, a reviewer suggested that we apply FDR correction which we provide for comparison with our preregistered PCA-based modification of Bonferroni correction.

#### Sensitivity analyses: EpiScore and cognitive ability associations, accounting for perinatal inflammatory exposures

Finally, to explore the role of inflammation in the observed associations, we conducted sensitivity analyses in the subgroup of preterm children, additionally accounting for the cumulative burden of perinatal inflammatory morbidities (HCA, sepsis, BPD and NEC). These exposures were treated as clinical indicators of perinatal inflammatory burden rather than confounders. The cumulative burden of perinatal inflammation, indexed by total number of these inflammatory exposures, was previously shown to be positively associated with DNAm CRP [28]. In baseline and adjusted models where inflammatory exposures were included as covariates, we quantified the degree of attenuation in the main effect of DNAm CRP or other EpiScores by comparing magnitude of the hierarchical standardised regression coefficients (β) and additional variance explained in the models (ΔR^2^). These analyses allowed us to distinguish variance captured by methylation-based proxies from that explained by documented inflammatory morbidities, quantifying the extent to which EpiScores show inflammation-independent associations with cognitive ability.

We also examined associations between cumulative perinatal inflammatory burden and 2-year Bayley-III cognitive composite or 5-year MSEL ELC, in univariable models, with and without adjustment for the EpiScores that showed significant associations with cognition. To assess whether these EpiScores reflected perinatal inflammatory states, we tested for differences of their distributions across number of perinatal inflammatory morbidities using Kruskal-Wallis test, followed by pairwise Wilcoxon rank-sum tests with FDR correction. Finally, logistic regression was used to examine associations between these EpiScores and individual morbidities (HCA, BPD, NEC or sepsis), adjusting for gestational age at sampling, infant sex and birthweight Z-score.

## Results

### Participant characteristics

At 2 years, *n* = 154 preterm children with neonatal EpiScores were assessed for Bayley-III, characteristics of whom are reported in Supplementary Table 2. At 5 years, 127 children (67 preterm) were assessed for MSEL, characteristics of whom are shown in Table 1. MSEL ELC scores tended to be lower in preterm children (Table 1).

**Table 1:** Demographic and clinical characteristics of participants with neonatal EpiScores and 5-year Mullen Scales of Early Learning (MSEL) data (*n* = 127).

	Term	Preterm	SMD
n	60	67	
Sex: Female, n (%)	24 (40.0%)	31 (46.3%)	0.127
Gestational age at birth / weeks, median (range)	39.64 (36.43, 42.14)	29.42 (25.14, 32.00)	6.796
Gestational age at DNAm sampling / weeks, median (range)	42.14 (40.00, 46.14)	40.57 (37.70, 43.43)	1.324
Neonatal DNAm CRP, median (range)	−0.011 (−0.012, −0.010)	−0.010 (−0.013, −0.010)	0.292
Chronological age at MSEL assessment / months, median (range)	60.5 (59.0, 63.9)	60.4 (59.4, 65.3)	0.152
MSEL Early Learning Composite, median (range)	97 (70, 121)	89 (60, 127)	0.349
Birthweight / g, median (range)	3454 (2410, 4560)	1112 (600, 2100)	6.264
Birthweight z-score, median (range)	0.45 (−2.30, 2.57)	−0.03 (−2.62, 1.58)	0.698
Histological chorioamnionitis, n (%)	3 (8.6)	16 (24.6)	0.442
Sepsis, n (%)	NA	14 (20.9)	–
Bronchopulmonary dysplasia, n (%)	NA	21 (32.3)	–
Necrotising enterocolitis, n (%)	NA	2 (3.0)	–
Maternal age / years, median (range)	33 (24, 47)	33 (20, 44)	0.283
Maternal BMI / median (range)	23.90 (18.20, 47.00)	25.10 (16.40, 48.00)	0.185
Smoked during pregnancy, n (%)	0 (0.0)	6 (9.1)	0.447
Corticosteroid administration in pregnancy, n (%)	0 (0.0)	64 (95.5)	6.532
MgSO_4_ administration in pregnancy, n (%)	NA	50 (74.6)	–
Mother university degree, n (%)	54 (90.0)	42 (62.7)	0.679
SIMD rank, median (range)	5471 (727, 6951)	4475 (137, 6929)	0.461

For categorical data, Chi-square test was used to compare proportions. For continuous data, Mann-Whitney *U-*test was used. Standardised mean difference, SMD, is derived from Cohen’s *d* for continuous data. Values are denoted as n (%) or median (range).

*SIMD* scottish index of multiple deprivation.

### Associations between DNAm CRP and general cognitive abilities

DNAm CRP was not associated with 2-year Bayley-III Cognitive composite in preterm children (*n* = 150) in univariable analyses (β = 0.014 [90% CI −0.122, 0.150], *p* = 0.862) or in baseline models adjusted for gestational age at sampling, sex and processing batch (β = 0.004 [90% CI −0.159, 0.167], *p* = 0.967), or models adjusted for additional prematurity-related and socioeconomic variables (β = 0.024 [90% CI −0.139, 0.187], *p* = 0.807; Supplementary Table 3).

**Fig. 2:**
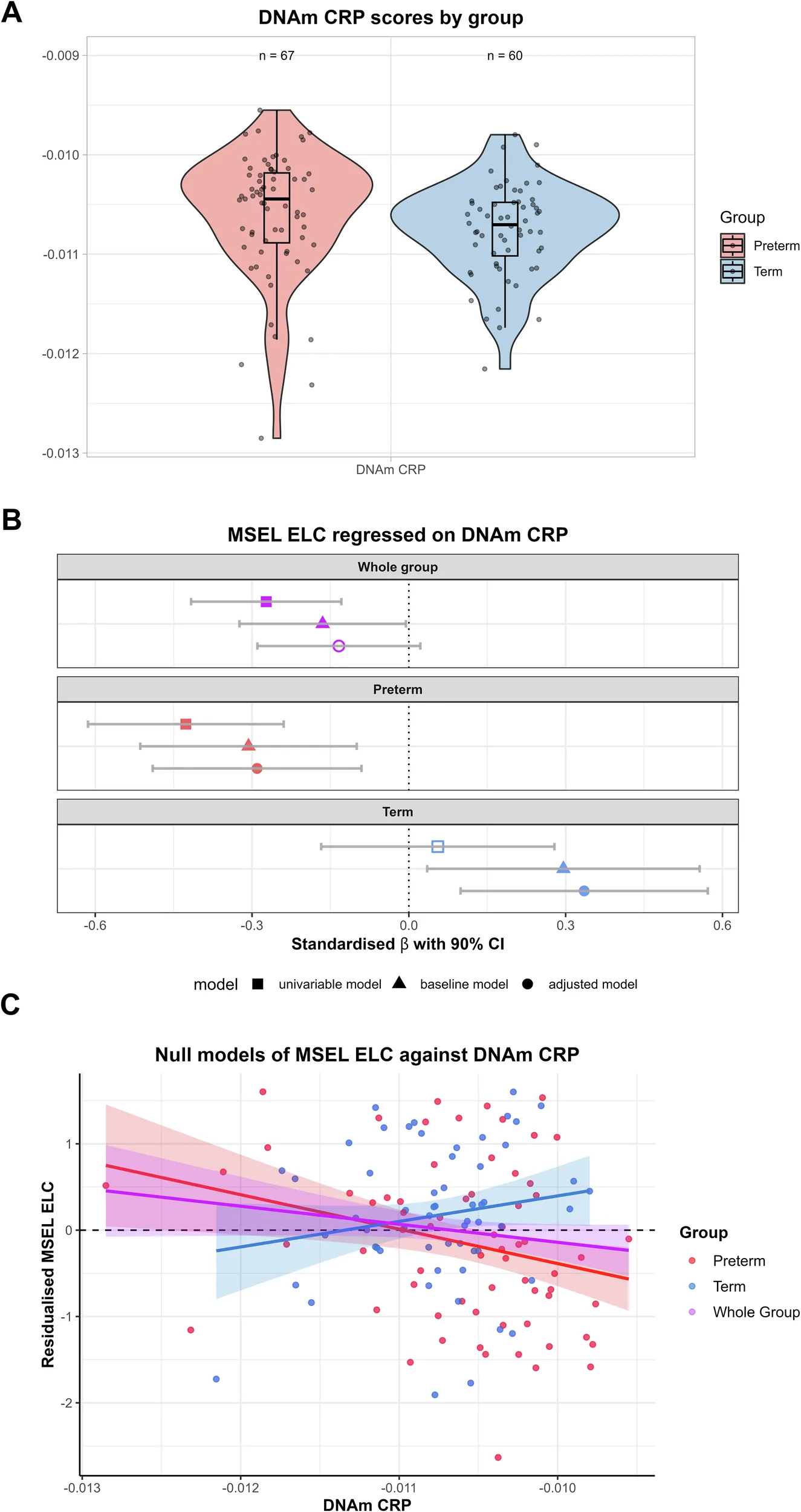
Neonatal DNAm CRP in preterm and term-born children and its association with MSEL Early Learning Composite (ELC), illustrating Simpson’s paradox. **A** Violin and box plots of DNAm CRP by group. **B** Forest plots for DNAm CRP associations with MSEL ELC for whole group, preterm and term-born children. Points show standardised regression coefficients and 90% confidence intervals (tests of specific, directional, pre-registered hypotheses). Significant associations are denoted by filled shapes, where *p* < 0.10. Baseline models are controlled for infant sex, gestational age at sampling and batch. Adjusted models additionally control for gestational age at birth, birthweight Z-score, maternal university education and SIMD. **C** Scatterplots with regression lines by gestational age, conveying Simpson’s paradox. Residuals from null models (adjusted for infant sex, gestational age at sampling and batch) are plotted against DNAm CRP. MSEL mullen scales of early learning, SIMD scottish index for multiple deprivation.

**Fig. 3:**
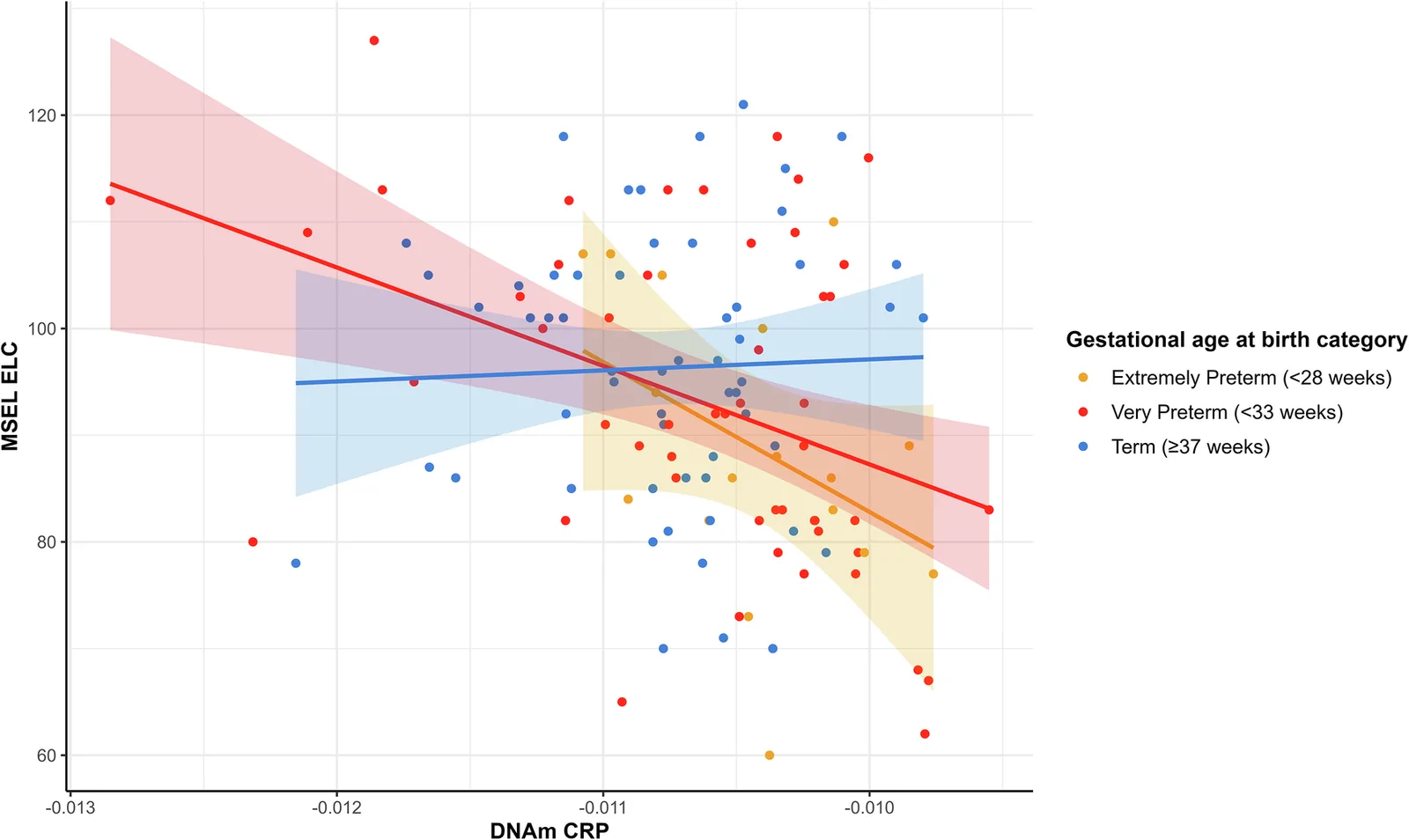
Interaction effects of DNAm CRP and gestational age on MSEL Early Learning Composite (ELC) at five years of age. Interactions are illustrated as three groups for ease of interpretation, namely extremely preterm, very preterm and term.

Median neonatal DNAm CRP was significantly higher in preterm compared to term-born children in those with 5-year MSEL ELC data (SMD = 0.292; Table 1; Fig. 2A). Higher DNAm CRP was associated with lower 5-year MSEL ELC scores in whole group analyses (*n* = 125) in univariable models (β = −0.273 [90% CI −0.147, −0.129], *p* = 0.002) and baseline models (β = −0.165, [90% CI −0.324, −0.006], *p* = 0.089), consistent with the directional hypothesis at *p* < 0.10 (Fig. 2B). However, magnitude of associations between DNAm CRP and MSEL ELC were reduced to non-significance after adjusting for gestational age at birth and socioeconomic exposures (absolute percentage decrease in β = 3%; *p* = 0.157; Fig. 2B), suggesting Simpson’s paradox where gestational age may be either a confounder or an interaction term [53]. Indeed, whole group analyses revealed significant interaction effects between gestational age and DNAm CRP in baseline models (product interaction term β = 0.181; main effect β = −0.034) and adjusted models (product interaction term β = 0.240; main effect β = −0.064), indicating that at lower gestational ages, the negative effect of DNAm CRP on MSEL ELC is stronger (Fig. 3; Supplementary Table 5).

The interaction effect was further supported by subgroup analyses (Fig. 2B; Supplementary Table 4). Specifically, in the preterm group (*n* = 67), higher DNAm CRP was consistently associated with lower MSEL ELC scores across all models: univariable (β = −0.427 [90% CI −0.614, −0.239], *p* < 0.001), baseline (β = −0.307 [90% CI −0.514, −0.100], *p* = 0.016) and fully adjusted (β = −0.290 [90% CI −0.490, −0.091], *p* = 0.018). In contrast, among term-born children (*n* = 58), univariable models showed no association (β = 0.055, *p* = 0.680), whereas inclusion of covariates in baseline and adjusted models revealed a positive association (β = 0.296 and 0.335, respectively; *p* < 0.10). This positive association appeared to be driven by adjustment for processing batch (batch β = −1.167, *p* = 0.001) as no other covariates showed significant associations; notably, batch was not correlated with other covariates suggesting it acted as a suppressor variable.

### Associations between DNAm CRP and domain-specific cognitive abilities

Given that DNAm CRP was associated with global cognitive ability at 5 years, we investigated whether associations are domain-specific using MSEL subscales (Supplementary Fig. 4). Higher DNAm CRP was associated with lower MSEL score across most subscales in univariable analyses for the whole group (β = −0.203 to −0.243, p_FDR_ < 0.05; Supplementary Table 6) and preterm subgroup (β = −0.258 to −0.485, p_FDR_ < 0.05; Supplementary Table 7). Further, associations in preterm children remained significant for fine motor (β = −0.284 to −0.314, p_FDR_ < 0.05) and visual reception (β = −0.396 to −0.437, p_FDR_ = < 0.05) in baseline and adjusted models. Comparing magnitudes of DNAm CRP associations across domains in preterm baseline models revealed significant differences between visual reception and both receptive language (t = −3.143, *p* = 0.002) and expressive language (t = −2.602, *p* = 0.009); no differences were observed between other domains (Supplementary Table 8).

### Associations between other EpiScores and general cognitive abilities in children born across the gestational age range

**Fig. 4:**
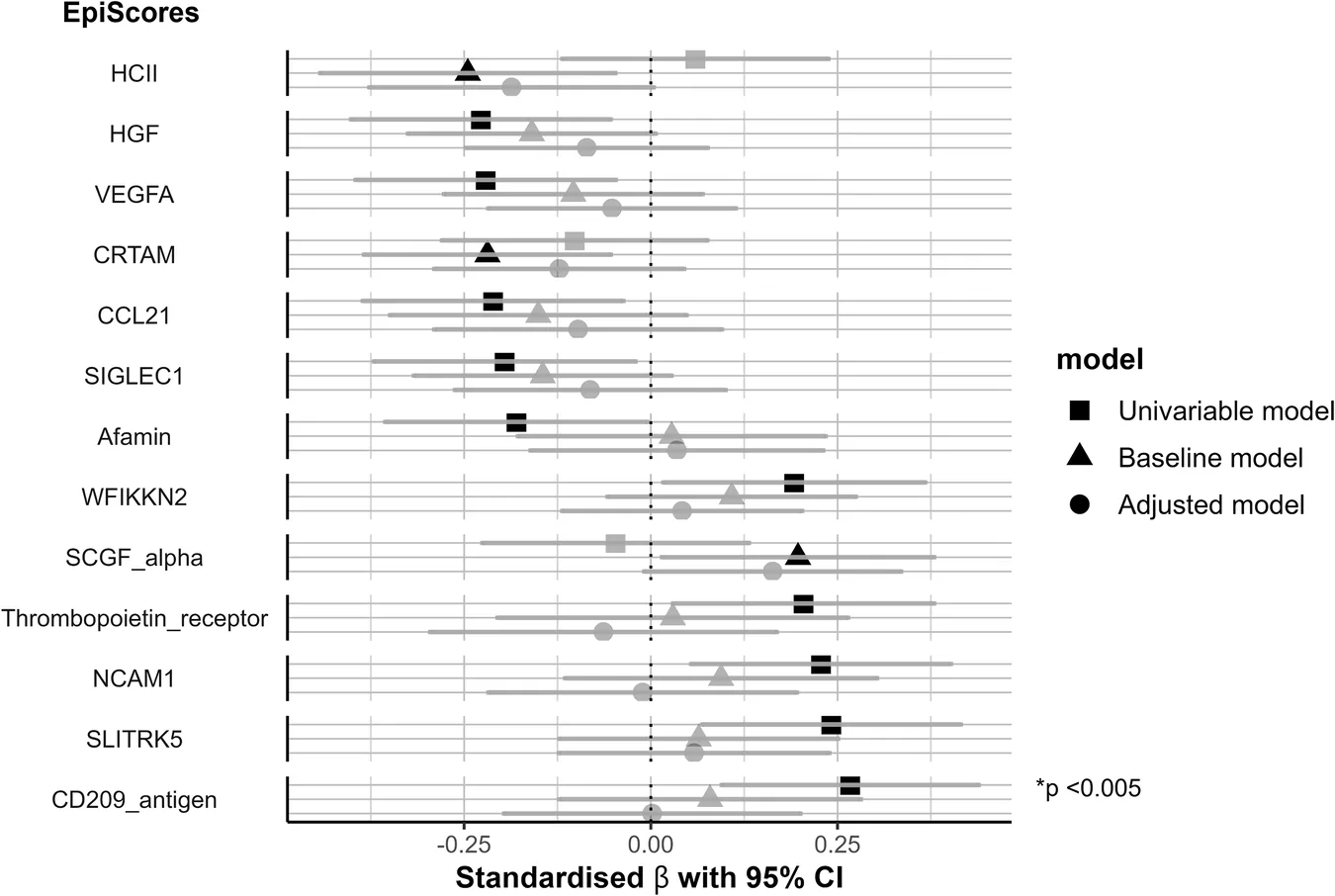
Association between EpiScores with MSEL Early Learning Composite. Points show standardised regression coefficients and 95% confidence intervals. Significant associations at the nominal threshold *p* < 0.05 are denoted by black shapes. Asterisks (*) indicate significant associations at the adjusted threshold *p* < 0.005. Baseline models are controlled for infant sex, gestational age at sampling and batch. Adjusted models additionally control for gestational age at birth, birthweight Z-score, maternal university education and SIMD. Models showing significant associations are ordered by magnitude. MSEL mullen scales of early learning, SIMD scottish index for multiple deprivation.

DNAm CD209 (β = 0.267 [95% CI 0.095, 0.439], *p* = 0.003) was positively associated with 5-year MSEL ELC, meeting the adjusted *p*-value threshold of < 0.005, in univariable analyses of the full cohort. Five other EpiScores were negatively (β = −0.178 to −0.228) and four positively associated (β = 0.192 to 0.242) with 5-year MSEL ELC scores in univariable analyses, but these nominal associations did not survive correction for multiple comparisons (*p*-values ≤ 0.045, with adjusted *p*-value threshold < 0.005; Fig. 4; Supplementary Table 9). In baseline models covarying for gestational age at sampling, sex and processing batch, DNAm CRTAM (β = −0.219 [95% CI −0.384, −0.054], *p* = 0.01) and DNAm HCII (β = −0.245 [95% CI −0.443, −0.048], *p* = 0.015) were negatively and DNAm SCGF alpha (β = 0.197 [95% CI 0.015, 0.379], *p* = 0.034) was positively associated with MSEL ELC at the uncorrected threshold. No EpiScore associations survived additional adjustment for prematurity-related or socioeconomic variables in whole group analyses. Associations identified using the pre-registered PCA-based approach to correct for multiple testing did not withstand Benjamini-Hochberg FDR correction (p_FDR_ ≥ 0.06; Supplementary Tables 9–11).

### Associations between EpiScores and cognition in preterm children, accounting for perinatal inflammatory exposures

Finally, in sensitivity analyses, we tested the hypothesis that for preterm children (*n* = 67), exposure to perinatal inflammatory events partly accounts for the association between EpiScores and poorer cognitive outcomes. Main effect size of association between DNAm CRP and 5-year MSEL ELC remained statistically significant and unattenuated after controlling for number of perinatal inflammatory exposures in preterm children (β = −0.318 [95% CI −0.543, −0.093], absolute percentage β increase = 1.1%, *p* = 0.021; Table 2), with only a marginal increase in explained variance (ΔR^2^ = 0.1%).

**Table 2:** Associations between DNAm CRP and MSEL Early Learning Composite, adjusting for number of inflammatory exposures in preterm children only.

model	Beta	Lower CI	Upper CI	p	R^2^	Incremental R^2^	n
baseline model	**−0.307**	−0.514	−0.100	**0.016**	0.263	0.060	67
baseline model + inflammatory exposures	**−0.318**	−0.543	−0.093	**0.021**	0.264	0.061	65
adjusted model + inflammatory exposures	**−0.323**	−0.532	−0.113	**0.013**	0.400	0.197	63

Betas (standardised regression coefficients for DNAm CRP) and 90% confidence intervals are reported. Incremental R^2^ refers to the amount of variance in MSEL ELC accounted for by DNAm CRP (and other inflammatory, prematurity-related or socioeconomic exposures), beyond that of baseline covariates: sex, gestational age at sampling and batch. Bold text denotes *p* < 0.10. Baseline model = MSEL ELC ~ DNAm CRP + sex + gestational age at sampling + batch Adjusted model = MSEL ELC ~ DNAm CRP + sex + gestational age at sampling + batch + gestational age at birth + birthweight Z score + SIMD + maternal university education.

Bold text denotes *p* < 0.10.

DNAm CCL18 (β = 0.198 [95% CI 0.036, 0.361], *p* = 0.017) and DNAm CD5L (β = 0.238 [95% CI 0.016 to 0.460], *p* = 0.036) were nominally associated with 2-year Bayley-III Cognitive composite in preterm children in baseline and adjusted models. Associations for DNAm CCL18 additionally remained significant after adjustment for inflammatory exposures (absolute percentage decrease in β = 1.6% [95% CI 0.009, 0.354], *p* = 0.039, ΔR^2^ = 0.1%; Supplementary Table 10).

DNAm CRTAM (β = −0.307 [95% CI −0.523, −0.090], *p* = 0.006) and DNAm NCAM1 (β = 0.294 [95% CI 0.013 to 0.574], *p* = 0.040) were nominally associated with 5-year MSEL ELC in preterm children in baseline and adjusted models. Associations for DNAm CRTAM additionally remained significant after adjustment for inflammatory exposures (absolute percentage change in β = 0% [95% CI −0.531, −0.083], *p* = 0.008, ΔR^2^ = 0.3%; Supplementary Table 11).

Perinatal inflammatory burden itself was not associated with 2-year Bayley-III cognitive composite (β = 0.054 [95% CI −0.115, 0.224], *p* = 0.526, R^2^ = 0.003) or 5-year MSEL ELC (β = 0.008 [95% CI −0.253, 0.268], *p* = 0.953, R^2^ < 0.001) in univariable analyses. This lack of association remained after including DNAm CCL18 in the Bayley-III model (inflammatory burden: β = 0.012 [95% CI −0.007, 0.332], *p* = 0.883, ΔR^2^ = 2.6%). Likewise, inclusion of DNAm CRP in the MSEL model did not alter the null association between inflammatory burden and cognition (β = 0.017 [95% CI −0.215, 0.250], *p* = 0.882, ΔR^2^ = 21.3%). Similar results were observed after inclusion of DNAm CRTAM (inflammatory burden: β = 0.033 [95% CI −0.221, 0.287], *p* = 0.797, ΔR^2^ = 7.2%).

Associations of higher DNAm CRP with increased odds of HCA, BPD, NEC and sepsis, as well higher DNAm CRP in infants with greater numbers of inflammatory episodes have been previously reported [28]. Similarly, in this sample of preterm infants (*n* = 171), DNAm CCL18 was higher in infants who experienced more inflammatory episodes (Supplementary Table 12) and was associated with increased odds of BPD (OR = 1.612 [95% CI 1.196, 2.211], *p* = 0.002) and sepsis (OR = 1.790 [95% CI 1.298, 2.526], *p* < 0.001; Supplementary Table 13). In contrast, DNAm CRTAM showed no association with cumulative inflammatory burden (Supplementary Table 14) or with the odds of experiencing most individual exposures, with the exception of BPD (OR = 1.414 [95% CI 1.053, 1.919], *p* = 0.023; Supplementary Table 15).

## Discussion

We leveraged summary statistics from large-scale EWAS and penalised regression models to calculate methylation-based biomarkers, EpiScores, in neonatal saliva and integrated these with cognitive outcome data at two timepoints in childhood to investigate relations between chronic low-grade systemic perinatal inflammation and later cognition. Our key, novel finding was that DNAm CRP and DNAm CD209 are significantly associated with general cognition at age 5 years, with an additional fourteen demonstrating nominal (uncorrected) significance at either 2 or 5 years. We showed that higher neonatal DNAm CRP associated with poorer 5-year MSEL Early Learning Composite, with moderate effect size, β = −0.163 to −0.307, the strength of which is more pronounced with decreasing gestational age at birth. Associations of DNAm CRP, DNAm CCL18 and DNAm CRTAM in preterm children were independent of the number of inflammatory exposures, suggesting unique contributions of specific immuno-epigenetic signatures to variance in cognition beyond immune-related morbidities.

Given previously established associations of neonatal DNAm CRP with gestational age at birth, perinatal inflammatory exposures and MRI features of EoP [27, 28], we examined the relation of this marker with cognition in this study separately to the other EpiScores which are associated with gestational age at birth. The observation that higher neonatal DNAm CRP associates with lower cognitive score in 5-year-olds is consistent with findings that blood-derived inflammation-related epigenetic polygenic risk scores are associated with childhood cognition [30]. Here, we show CRP EpiScore derived using non-invasive sampling methods is associated with cognition at school age, and that the magnitude of effect is dose-dependent with degree of prematurity. This finding aligns with previous observations of elevated inflammatory protein concentrations, including CRP, in early life associating with cognition in extremely preterm infants [6, 54], lending support to the clinical value of this methylation-based proxy. The analogous protein is an acute-phase reactant in the immune response, but the mechanisms through which it may impact cognition remain unclear. Notably, DNAm CRP was not associated with 2-year cognition according to the Bayley-III, which could be a function of test performance leading to overestimation of cognitive ability [55, 56]. Consistent with this, among preterm children with outcome data available at both timepoints, cognitive scores at 2 and 5 years were only moderately correlated in our cohort (r = 0.55), suggesting that EpiScores may be more strongly related to later-emerging, stable cognitive function. Alternatively, DNAm CRP and most other protein EpiScores may not be sufficiently sensitive to detect differences in earlier stages of cognitive development.

Follow-up analyses with 5-year MSEL subscales revealed negative associations between DNAm CRP and all domains, with magnitudes largest for fine motor and visual reception in preterm children, even after adjusting for prematurity-related and socioeconomic factors. Williams test [50] confirmed that visual reception was disproportionately associated with DNAm CRP compared to language domains. The visual reception MSEL subscale assesses visual information processing, which depends on unimpaired development of ocular and visual cognitive functions. Whereas previous studies demonstrate associations between serum CRP and development of retinopathy of prematurity (ROP) [57, 58], a hallmark preterm morbidity leading to visual impairment, our previous work showed no independent association between DNAm CRP at term-equivalent age and higher odds of requiring treatment for ROP in this sample [28]. An alternative explanation is that neonatal DNAm CRP is associated with cerebral visual impairment, which is common among preterm-born school children and thought to be related to weaker structural connectivity in visual networks secondary to diffuse white matter dysmaturation [59]. Thus, the relation between inflammatory burden of prematurity captured by neonatal DNAm CRP and cerebral visual function warrants further investigation, particularly since visual processing is important for development of language, motor and other skills in early life.

In addition to DNAm CRP, we found suggestive evidence that other EpiScores may be associated with cognition in children born across the gestational age range. These associations were not fully consistent across timepoints and models, which may reflect developmental variability in early cognition and heterogeneity across the full gestational age spectrum, rather than absence of physiological relevance. Notably, the analogous proteins implicated by these EpiScores converge on biologically plausible pathways, including the immune response (HCII, CRTAM), neurogenesis (NCAM1, SLITRK5), vascular development (VEGFA), or are growth factors (HGF, SIGLEC1, WFIKKN2, SCGF alpha), chemokines (CCL21) or cell membrane proteins (Thrombopoietin receptor, CD209, Afamin). VEGFA, in particular, was previously indicated in the extremely low gestational age newborn cohort to associate with neurodevelopmental outcomes [6]. Although the EpiScores themselves have not been previously studied in a neurodevelopmental context, DNAm NCAM1 and DNAm SLITRK5, both positively associated with 5-year cognition in this study, were associated with slower time-to-dementia in an adult cohort [29]. Their analogous proteins are localised at synaptic sites and play established roles in neuronal growth and synaptic plasticity [60, 61]. Most associations did not survive correction for multiple comparisons and other exposures in this moderately-sized sample, possibly reflecting the highly correlated structure of the protein EpiScores together with a modest enrichment of small *p*-values. However, the nominal associations identify biologically plausible candidates that warrant replication in independent cohorts to assess the generalisability of the findings across diverse samples.

In contrast, in analyses restricted to preterm children, the EpiScores for CRP, CRTAM and CCL18 remained nominally significant and consistent in direction across multiple model specifications, including after adjustment for prematurity-related, socioeconomic and inflammatory exposures. Indeed, accounting for morbidities did not considerably increase the explained variance in cognition, suggesting a unique, more substantial contribution of the EpiScores to variance in cognition over residual inflammatory experience. This is consistent with our previous study [28] showing similarly robust, independent associations of DNAm CRP with white matter microstructure, and highlights CRTAM and CCL18 as other notable target biomarkers in this population. Low cord blood levels of the chemokine CCL18 have previously been linked to an increased risk of cerebral palsy in preterm children [62] and, while not previously studied in the context of preterm birth, *CRTAM* was recently identified as an immune-related Alzheimer’s disease risk gene using post mortem tissue [63].

Interestingly, perinatal inflammatory burden was not associated with cognitive outcomes. This may reflect limited statistical power for detecting clinical antecedents of complex functional traits such as cognition, or heterogeneity in type and timing of exposures among participants. An alternative account is that protein EpiScores may better reflect integrated downstream molecular consequences of inflammation on neurodevelopment. CRTAM, CRP and CCL18 EpiScores explain up to 11% of variance in their respective inflammatory proteins [16] and correlate with inflammatory morbidities in this cohort, supporting their relevance to inflammatory processes. At the same time, given that they are based on epigenetic variation, these protein EpiScores may capture broader molecular signals relevant to not only inflammation, but also prematurity [27] and neurodevelopment through their constituent CpGs. This may explain the stability of associations observed in the preterm subgroup after adjustment for gestational age and birthweight. Such molecularly integrated signal may therefore explain why EpiScores for CRP, CRTAM and CCL18 were more ‘predictive’ of cognitive outcomes than the raw count of inflammatory exposures. Nevertheless, as noted by Schalkwijk et al. [64], determining the added predictive value of protein EpiScores for neurocognitive outcomes will require direct comparisons with measured protein concentrations.

Many protein EpiScores in this study were previously found to associate with MRI-derived brain volumes in adulthood, both in cognitively normal and at-risk adult cohorts [21, 29]. Various MRI metrics also partly mediated DNAm CRP-cognition association, with white matter volume contributing most strongly [21]. We previously showed that in preterm neonates, DNAm CRP associated with regional and global brain volumes, and most strongly with white matter microstructure [28]. In a predominantly term cohort, DNAm CRP derived in cord blood has similarly been associated with white matter development [32]. It may be that the particular vulnerability in preterm children to widespread brain alterations is augmented by inflammatory experience, which in turn contributes to poorer childhood cognition [65]. Sustained elevation of inflammatory proteins in extremely preterm infants was demonstrated to associate with lower IQ at age 10, even after adjusting for various brain volume metrics [8]. In another study, inflammatory events predicted alterations in corpus callosum microstructure, which correlated with cognitive performance at age 6 [66]. Given that protein EpiScores outperform proteomic associations with brain structure [21, 23] and are associated with childhood cognition, future work could investigate whether white matter microstructure mediates EpiScore-cognition associations. Several protein EpiScores identified here may be indicative of disrupted neurodevelopmental processes including neurogenesis, synaptogenesis and myelination [13, 65].

Strengths of this study include the range of multimodal data allowing us to profile numerous epigenetic biomarkers and characterise cognition at multiple time points, as well as account for relevant inflammatory, prematurity-related and socioeconomic factors. Particularly in this population, use of salivary-based methylation proxies of inflammatory proteins offers a non-invasive, more stable, attractive alternative to blood sampling of proteins in neonates. The shared ectodermal origins of buccal and neural tissue as well as high correlation of DNA methylation patterns in both tissues (R = 0.85) support the use of salivary-based EpiScores for investigations regarding cognition [67]. In terms of limitations, application of EpiScores to age groups, tissue types, array platforms and ancestral backgrounds that differ from those of the original training dataset is a well-recognised challenge in the field of developmental epigenomics [68]. However, DNAm CRP has been validated against serum CRP in cord blood at birth [32] and adult-trained DNAm profile scores can be informative in paediatric samples: for example, in a study of 1183 youths (8–19 years), adult-derived salivary DNAm CRP and a DNAm proxy of cognitive performance were socially stratified and associated with multiple cognitive and academic outcomes [48]. Although further research is required to understand the applicability of adult-derived scores in younger populations, the correlation of these saliva-based protein EpiScores with gestational age, and DNAm CRP with perinatal morbidities, supports clinical salience in early life [27, 28]. While the prospective nature of our data offers the strength of longitudinal observations, our preterm-term subgroup analyses in particular may be limited by the modest sample size. Different assessments were conducted in different subgroups at 2 and 5 years, in partly overlapping samples; therefore, these timepoints provide complementary but not directly comparable information about cognitive outcomes across early childhood. In addition, while DNAm may offer greater temporal stability than protein measurement, it is dynamic in relation to development and ageing [69]. The present study does not address the temporal stability or developmental evolution of protein EpiScores, as DNAm was measured at a single neonatal time point. Longitudinal DNAm profiling across infancy and childhood will be required to establish the extent to which these EpiScores represent stable inter-individual differences versus developmentally dynamic signatures that track changing immune and developmental states [68, 70]. Nonetheless, in this study of preterm infants, single sampling at term-equivalent age is advantageous for capturing DNAm signatures of preterm birth and its co-exposures during neonatal intensive care. Finally, approaches such as Mendelian randomisation and preclinical studies are necessary to determine causality and isolate confounding genetic and environmental factors. Of interest would be the stability of these associations, given the sustained inflammation experienced by preterm infants, which itself is associated with neurocognitive outcomes [6, 71, 72]. Nonetheless, the present study provides essential discovery insights motivating work towards individual-level prediction, validation and replication in prognostic studies, where positive and negative predictive values of neonatal protein EpiScores for cognition can be estimated.

Overall, in a cohort of children spanning the whole gestational age range, we found evidence suggesting that neonatal methylation-based EpiScores were associated with general cognition at 2 or 5 years. In preterm children, associations were independent of perinatal inflammatory exposures. Our findings point to potential translational utility of peripheral methylation-based biomarkers for stratifying infants at risk of cognitive difficulties at preschool age.

## Supplementary information

Supplemental Material

## Data Availability

Anonymised data including EpiScores used in these analyses are deposited in Edinburgh DataVault (https://doi.org/10.7488/e65499db-2263-4d3c-9335-55ae6d49af2b) [73] and are available to researchers under the terms of the Theirworld Edinburgh Birth Cohort Data Access and Collaboration policy available at https://reproductive-health.ed.ac.uk/theirworld-edinburgh-birth-cohort-tebc/for-researchers/data-access-and-collaboration. Source code for primary analysis and figures are available at https://git.ecdf.ed.ac.uk/jbrl/neonatal-episcores-and-childhood-cognition.

## Code availability

Source code for primary analysis and figures are available at https://git.ecdf.ed.ac.uk/jbrl/neonatal-episcores-and-childhood-cognition.
