# Supplementary material for: Protein epigenetic scores derived in neonatal saliva as biomarkers of childhood cognition
Source: Mol Psychiatry. 2026 Jun 24;31(11):6327–38. doi: 10.1038/s41380-026-03702-4 (PMC13668716; doi:10.1038/s41380-026-03702-4)
Supplement: Supplementary file 1 [file 41380_2026_3702_MOESM1_ESM.docx]

**Protein epigenetic scores derived in neonatal saliva as biomarkers of childhood cognition**

Rebekah Smikle, PhD^1^, Katie Mckinnon, PhD^1^, Kadi Vaher, PhD^1,2^, Helen Turner, MBChB^2,3^, Hilary Cruickshank, BSc^3^, Ray Amir, MSc^1^, Yu Wei Chua, PhD^4^, Eleanor LS Conole, PhD^5^, Rebecca M Reynolds, PhD^2,6^, G David Batty, DSc^7^, Athanasios Tsanas, DPhil (Oxon)^8^, Lee Murphy, MSc^2,9^, Heather C Whalley, PhD^2^, Riccardo E Marioni, PhD^10^, Simon R Cox, PhD^11^, James P Boardman, PhD^1,2^

Affiliations

1. Centre for Reproductive Health, Institute for Regeneration and Repair, University of Edinburgh, Edinburgh, UK
2. Institute for Neuroscience and Cardiovascular Research, University of Edinburgh, Edinburgh, UK
3. Simpson Centre for Reproductive Health, Royal Infirmary Edinburgh, Edinburgh, UK
4. Department of Public Health, Policy and Systems, Institute of Population Health, University of Liverpool, Liverpool, UK
5. Department of Biochemistry, University of Oxford, Oxford, UK
6. Centre for Cardiovascular Science, Queen’s Medical Research Institute, University of Edinburgh, Edinburgh, UK
7. Department of Epidemiology and Public Health, University College London, London, UK
8. Usher Institute, Edinburgh Medical School, University of Edinburgh, Edinburgh, UK
9. Edinburgh Clinical Research Facility, University of Edinburgh, Western General Hospital, Edinburgh, UK
10. Institute of Genetics and Cancer, University of Edinburgh, Edinburgh, UK
11. Lothian Birth Cohorts, Department of Psychology, University of Edinburgh, Edinburgh, UK

**Correspondence**

James P. Boardman, Centre for Reproductive Health, Institute for Regeneration and Repair, University of Edinburgh, 4-5 Little France Drive, Edinburgh EH16 4UU, UK;
Email: [james.boardman@ed.ac.uk](mailto:james.boardman@ed.ac.uk)

**This document includes**

- Supplementary Figure 1. Flowchart of study sample from the neonatal period and data collection of the Bayley-III at 2 years and Mullen Scales of Early Learning (MSEL) at 5 years.
- Supplementary Figure 2. Principal component analysis of all 43 EpiScores to visualise DNAm processing batch effects.
- Supplementary Figure 3. Model diagnostic metrics for association of DNAm CRP with MSEL Early Learning Composite. Assumptions shown are tested for baseline model = MSEL ELC ~ DNAm CRP + sex + GA sampling + batch
- Supplementary Table 1. Loadings of the 42 EpiScores onto the first 10 principal components.
- Supplementary Table 2. Demographic and clinical characteristics of participants with neonatal EpiScores and 2-year Bayley Scales of Infant and Toddler Development (Bayley-III) data.
- Supplementary Table 3. Association of DNAm CRP with Bayley-III Cognitive Composite in preterm children at 2 years old.
- Supplementary Table 4. Association of DNAm CRP with MSEL Early Learning Composite (ELC) in preterm children and term-born children at 5 years old.
- Supplementary Table 5. Interaction effects between DNAm CRP and gestational age on MSEL Early Learning Composite in full cohort and preterm only. Standardised betas and p-values are reported from regression models where DNAm CRP is regressed onto MSEL ELC, with and without an interaction term, DNAm:CRP. Bold text denotes p <0.10.
- Supplementary Figure 4. Associations between DNAm CRP and MSEL subscales in whole group and preterm subgroup.
- Supplementary Table 6. Associations between DNAm CRP and MSEL subscales in exploratory analyses in the full cohort at 5 years old (n = 125).
- Supplementary Table 7. Associations between DNAm CRP and MSEL subscales in exploratory analyses in the preterm children only at 5 years old (n = 67).
- Supplementary Table 8**.** Williams’ test (1959) for differences in magnitudes of standardised DNAm CRP regression coefficients for baseline models between MSEL subscales: fine motor (FM), visual reception (VR), expressive language (EL), receptive language (RL).
- Supplementary Table 9. Association of EpiScores with MSEL Early Learning Composite (ELC) in the full cohort at 5 years old (n = 125).
- Supplementary Table 10. Association of EpiScores with Bayley-III Cognitive Composite in preterm children at 2 years old (n = 150).
- Supplementary Table 11. Associations between EpiScores and MSEL Early Learning Composite in preterm children only, adjusting for number of inflammatory exposures.
- Supplementary Table 12. Associations between DNAm CCL18 and number of inflammatory morbidities.
- Supplementary Table 13. Associations between DNAm CCL18 and individual perinatal inflammatory morbidities.
- Supplementary Table 14. Associations between DNAm CRTAM and number of inflammatory morbidities.
- Supplementary Table 15. Associations between DNAm CRTAM and individual perinatal inflammatory morbidities.

**Supplementary Figure 1**. Flowchart of study sample from the neonatal period and data collection of the Bayley-III at 2 years and Mullen Scales of Early Learning (MSEL) at 5 years. Of 171 preterm children, 104 had Bayley-III only, 17 had MSEL only and 50 had both.

**Supplementary Figure 2**. Principal component analysis of all 43 EpiScores to visualise DNAm processing batch effects.

**Supplementary Figure 3**. Model diagnostic metrics for association of DNAm CRP with MSEL Early Learning Composite. Assumptions shown are tested for baseline model = MSEL ELC ~ DNAm CRP + sex + GA sampling + batch

**Supplementary Table 1.** Loadings of the 42 EpiScores onto the first 10 principal components.

|  | PC1 | PC2 | PC3 | PC4 | PC5 | PC6 | PC7 | PC8 | PC9 | PC10 |
| --- | --- | --- | --- | --- | --- | --- | --- | --- | --- | --- |
| Afamin | 0.624 | -0.130 | -0.488 | -0.195 | 0.047 | -0.078 | 0.076 | 0.050 | 0.042 | 0.005 |
| CCL11 | 0.097 | -0.062 | 0.779 | -0.149 | 0.273 | 0.237 | 0.011 | -0.103 | -0.033 | -0.023 |
| CCL18 | 0.011 | 0.274 | -0.201 | 0.311 | -0.028 | -0.248 | 0.423 | -0.305 | 0.215 | 0.244 |
| CCL21 | 0.211 | 0.121 | 0.328 | -0.117 | -0.522 | 0.253 | 0.194 | -0.293 | 0.010 | 0.039 |
| CCL22 | 0.336 | -0.034 | 0.544 | -0.256 | 0.136 | 0.104 | 0.110 | 0.276 | 0.041 | -0.003 |
| CCL25C.C | 0.379 | -0.203 | -0.566 | -0.128 | 0.114 | -0.050 | 0.115 | 0.043 | 0.206 | 0.059 |
| CD163 | 0.440 | 0.715 | -0.081 | -0.052 | 0.101 | 0.223 | -0.079 | -0.024 | -0.001 | 0.033 |
| CD209_antigen | -0.858 | 0.153 | 0.194 | -0.012 | 0.065 | -0.013 | 0.073 | 0.006 | 0.078 | -0.019 |
| CD48_antigen | 0.437 | 0.788 | -0.186 | -0.072 | 0.035 | 0.018 | -0.077 | 0.101 | 0.004 | 0.063 |
| CD6 | 0.323 | 0.038 | 0.254 | -0.482 | 0.120 | 0.269 | 0.117 | -0.055 | -0.025 | -0.143 |
| CD5L | -0.494 | 0.610 | 0.245 | -0.255 | -0.036 | 0.155 | 0.002 | 0.004 | 0.022 | -0.045 |
| ComplementC9 | 0.376 | 0.062 | -0.069 | 0.207 | 0.326 | -0.057 | -0.157 | -0.205 | -0.414 | -0.309 |
| Contactin.4 | -0.460 | -0.213 | -0.534 | 0.194 | 0.289 | 0.253 | 0.182 | 0.062 | -0.116 | 0.157 |
| CXCL10_Olink | -0.131 | 0.693 | 0.109 | 0.219 | 0.021 | -0.078 | -0.004 | -0.087 | 0.046 | 0.160 |
| CXCL9 | 0.060 | 0.737 | -0.110 | 0.416 | -0.007 | -0.129 | -0.033 | 0.126 | 0.004 | -0.014 |
| CRTAM | -0.350 | 0.655 | 0.161 | 0.239 | -0.122 | 0.052 | -0.018 | 0.013 | -0.252 | -0.125 |
| FcRL2 | 0.462 | -0.049 | 0.057 | -0.460 | 0.218 | 0.295 | -0.139 | -0.060 | 0.076 | -0.151 |
| FGF.21 | 0.272 | 0.206 | 0.413 | -0.303 | -0.293 | -0.251 | -0.126 | 0.207 | -0.071 | -0.040 |
| GDF15 | 0.203 | 0.012 | -0.116 | 0.340 | -0.002 | -0.324 | 0.035 | -0.119 | -0.266 | -0.318 |
| GHR | -0.143 | -0.064 | -0.166 | -0.474 | 0.098 | -0.085 | -0.112 | -0.121 | -0.263 | 0.451 |
| HCII | 0.157 | -0.078 | 0.521 | 0.516 | 0.312 | -0.019 | -0.130 | -0.105 | -0.041 | 0.117 |
| HGF | 0.415 | 0.060 | 0.308 | 0.058 | -0.022 | -0.062 | 0.640 | 0.289 | -0.132 | 0.049 |
| IGFBP.1 | -0.313 | 0.019 | 0.194 | 0.126 | 0.308 | -0.263 | -0.105 | 0.426 | 0.289 | -0.270 |
| IGFBP.4 | 0.515 | 0.021 | 0.275 | -0.345 | -0.174 | -0.149 | -0.267 | -0.098 | 0.108 | -0.116 |
| L.selectin | -0.725 | 0.143 | 0.110 | 0.321 | -0.057 | 0.022 | 0.073 | 0.101 | 0.009 | -0.013 |
| Lactotransferrin | -0.150 | -0.079 | -0.023 | -0.020 | -0.301 | 0.184 | -0.191 | 0.319 | -0.537 | 0.292 |
| FCGR3A | 0.389 | 0.800 | -0.158 | 0.038 | 0.084 | 0.114 | -0.104 | 0.071 | 0.055 | 0.105 |
| MMP.9 | 0.501 | -0.171 | 0.487 | 0.458 | 0.062 | 0.011 | 0.024 | 0.081 | 0.018 | 0.186 |
| NCAM1 | -0.741 | 0.097 | 0.177 | -0.294 | 0.043 | 0.176 | 0.068 | 0.113 | 0.161 | 0.012 |
| PIGR | 0.319 | -0.090 | 0.836 | 0.183 | 0.099 | 0.101 | -0.031 | -0.002 | 0.041 | 0.112 |
| FAP | -0.353 | 0.196 | 0.244 | 0.147 | -0.161 | 0.113 | 0.320 | -0.376 | -0.126 | -0.270 |
| S100.A9 | -0.329 | 0.013 | -0.003 | 0.470 | -0.086 | 0.147 | -0.078 | 0.244 | -0.036 | -0.019 |
| SCGF alpha | 0.347 | -0.181 | -0.333 | 0.517 | -0.236 | 0.477 | -0.054 | 0.058 | 0.159 | -0.073 |
| SCGF beta | 0.449 | -0.185 | -0.372 | 0.411 | -0.271 | 0.443 | -0.076 | 0.100 | 0.187 | -0.087 |
| Semaphorin.3E | -0.609 | 0.133 | -0.411 | -0.395 | -0.149 | -0.012 | 0.075 | -0.023 | 0.099 | -0.119 |
| SKR3 | 0.246 | -0.065 | 0.455 | 0.295 | 0.291 | 0.011 | -0.002 | -0.125 | 0.166 | 0.145 |
| SIGLEC1 | 0.508 | 0.611 | -0.214 | -0.041 | 0.117 | -0.001 | 0.038 | 0.011 | 0.076 | -0.002 |
| SLITRK5 | -0.782 | 0.202 | 0.040 | -0.099 | 0.113 | -0.105 | 0.037 | 0.137 | 0.027 | -0.040 |
| Thrombopoietin receptor | -0.679 | 0.127 | 0.378 | -0.100 | -0.131 | 0.255 | 0.034 | 0.018 | 0.059 | -0.024 |
| Trypsin.2 | 0.269 | 0.087 | 0.389 | 0.031 | -0.596 | -0.333 | -0.185 | -0.022 | 0.217 | 0.122 |
| VCAM1 | 0.189 | 0.802 | -0.215 | -0.127 | 0.181 | 0.109 | -0.027 | -0.032 | 0.015 | 0.036 |
| VEGFA | 0.596 | 0.011 | 0.046 | -0.254 | 0.044 | -0.048 | 0.495 | 0.218 | -0.111 | -0.004 |
| WFIKKN2 | -0.526 | -0.067 | -0.025 | -0.032 | 0.266 | 0.020 | -0.158 | -0.206 | 0.125 | 0.210 |

**Supplementary Table 2**. Demographic and clinical characteristics of participants with neonatal EpiScores and 2-year Bayley Scales of Infant and Toddler Development (Bayley-III) data.

| n | 154 |
| --- | --- |
| Sex: Female, n (%) | 73 (47.4) |
| Gestational age at birth / weeks, median (range) | 29.21 (22.14, 32.84) |
| Gestational age at DNAm sampling / weeks, median (range) | 40.57 (36.57, 45.86) |
| Neonatal DNAm CRP, median (range) | -0.011 (-0.013, -0.010) |
| Corrected age at Bayley-III assessment / months, median (range) | 24.2 (18.2, 30.2) |
| Bayley-III Cognitive composite, median (range) | 110 (20, 145) |
| Birthweight / g, median (range) | 1150 (370, 2510) |
| Birthweight z-score, median (range) | 0.11 (-3.13, 2.14) |
| Histological chorioamnionitis, n (%) | 43 (30.1) |
| Sepsis, n (%) | 34 (22.1) |
| Bronchopulmonary dysplasia, n (%) | 47 (30.5) |
| Necrotising enterocolitis, n (%) | 3 (1.9) |
| Maternal age / years, median (range) | 32 (17, 44) |
| Maternal BMI, median (range) | 25.30 (16.40, 46.60) |
| Smoked during pregnancy, n (%) | 20 (13.1) |
| Corticosteroid administration in pregnancy, n (%) | 147 (95.5) |
| MgSO_4_ administration in pregnancy, n (%) | 108 (70.1) |
| Mother university degree, n (%) | 85 (55.2) |
| SIMD rank, median (range) | 4043 (14, 6966) |

SIMD = Scottish Index of Multiple Deprivation.

**Supplementary Table 3.** Association of DNAm CRP with Bayley-III Cognitive Composite in preterm children at 2 years old.

| **model** | **Beta** | **Lower CI** | **Upper CI** | **p** | **R^2^** | **n** |
| --- | --- | --- | --- | --- | --- | --- |
| univariable model | 0.014 | -0.122 | 0.150 | 0.862 | -0.007 | 150 |
| baseline model | 0.004 | -0.159 | 0.167 | 0.967 | 0.025 | 150 |
| adjusted model | 0.024 | -0.139 | 0.187 | 0.807 | 0.067 | 150 |

Betas (standardised regression coefficients for DNAm CRP) and 90% confidence intervals are reported. Baseline models are controlled for infant sex, gestational age at sampling and batch. Adjusted models additionally control for gestational age at birth, birthweight Z-score, maternal university education and SIMD.

**Supplementary Table 4.** Association of DNAm CRP with MSEL Early Learning Composite (ELC) in preterm children and term-born children at 5 years old.

| **group** | **model** | **Beta** | **Lower CI** | **Upper CI** | **p** | **R^2^** | **n** |
| --- | --- | --- | --- | --- | --- | --- | --- |
| Whole group | univariable model | **-0.273** | -0.417 | -0.129 | **0.002** | 0.067 | 125 |
|  | baseline model | **-0.165** | -0.324 | -0.006 | **0.089** | 0.177 | 125 |
|  | adjusted model | -0.134 | -0.290 | 0.022 | 0.157 | 0.281 | 125 |
| Preterm | univariable model | **-0.427** | -0.614 | -0.239 | **0.000** | 0.169 | 67 |
|  | baseline model | **-0.307** | -0.514 | -0.100 | **0.016** | 0.263 | 67 |
|  | adjusted model | **-0.290** | -0.490 | -0.091 | **0.018** | 0.389 | 67 |
| Term | univariable model  baseline model  adjusted model | 0.055 | -0.168 | 0.279 | 0.680 | -0.015 | 58 |
|  |  | **0.296** | 0.035 | 0.556 | **0.063** | 0.184 | 58 |
|  |  | **0.335** | 0.099 | 0.572 | **0.021** | 0.352 | 58 |

Betas (standardised regression coefficients for DNAm CRP) and 90% confidence intervals are reported. Bold text indicate statistically significant associations (p < 0.10). Baseline models are controlled for infant sex, gestational age at sampling and batch. Adjusted models additionally control for gestational age at birth, birthweight Z-score, maternal university education and SIMD.

**Supplementary Table 5.** Interaction effects between DNAm CRP and gestational age on MSEL Early Learning Composite in full cohort and preterm only. Standardised betas and p-values are reported from regression models where DNAm CRP is regressed onto MSEL ELC, including an interaction term (DNAm CRP x gestational age at birth (GA birth)), in the model. Bold text denotes p <0.10.

| **group** | **model** | **Effect** | **Beta** | **Lower CI** | **Upper CI** | **p** | **R^2^** | **n** |
| --- | --- | --- | --- | --- | --- | --- | --- | --- |
| Whole group | baseline model | DNAm CRP x GA birth | **0.181** | 0.029 | 0.332 | **0.050** | 0.243 | 125 |
|  |  | DNAm CRP | -0.034 | -0.200 | 0.131 | 0.733 | 0.243 | 125 |
|  | adjusted model | DNAm CRP x GA birth | **0.240** | 0.093 | 0.386 | **0.008** | 0.319 | 125 |
|  |  | DNAm CRP | -0.064 | -0.221 | 0.094 | 0.504 | 0.319 | 125 |
| Preterm | baseline model | DNAm CRP x GA birth | -0.055 | -0.262 | 0.153 | 0.662 | 0.335 | 67 |
|  |  | DNAm CRP | **-0.226** | -0.430 | -0.022 | **0.069** | 0.335 | 67 |
|  | adjusted model | DNAm CRP x GA birth | -0.034 | -0.247 | 0.179 | 0.791 | 0.379 | 67 |
|  |  | DNAm CRP | **-0.285** | -0.489 | -0.081 | **0.023** | 0.379 | 67 |

Baseline model = MSEL ELC ~ DNAm CRP*GA birth + sex + GA sampling + batch
Adjusted model = MSEL ELC ~ DNAm CRP* GA birth + sex + GA sampling + batch + birthweight Z score + SIMD + maternal university education

**Supplementary Figure 4.** Associations between DNAm CRP and MSEL subscales in whole group and preterm subgroup. Standardised regression coefficients are shown, where points show standardised coefficients and 95% confidence intervals. Significant associations are denoted by filled shapes corrected for false discovery rate (where q <0.05). Baseline models are controlled for infant sex, gestational age at sampling and batch. Adjusted models additionally control for gestational age at birth, birthweight Z-score, maternal university education and SIMD.

**Supplementary Table 6.** Associations between DNAm CRP and MSEL subscales in exploratory analyses in the full cohort at 5 years old (n = 125). Betas (standardised regression coefficients for DNAm CRP) and 95% confidence intervals are reported. Bold text indicate statistically significant associations which survive FDR correction for multiple comparisons (q <0.05).

| **MSEL subscale** | **model** | **Beta** | **Lower CI** | **Upper CI** | **p** | **R^2^** | **q** |
| --- | --- | --- | --- | --- | --- | --- | --- |
| Fine Motor | Univariable model | **-0.238** | -0.411 | -0.064 | 0.008 | 0.049 | **0.015** |
|  | Baseline model | -0.188 | -0.377 | 0.001 | 0.052 | 0.188 | 0.175 |
|  | Adjusted model | -0.127 | -0.314 | 0.060 | 0.182 | 0.271 | 0.365 |
| Visual Reception | Univariable model | **-0.243** | -0.416 | -0.070 | 0.006 | 0.052 | **0.015** |
|  | Baseline model | -0.175 | -0.377 | 0.026 | 0.088 | 0.076 | 0.175 |
|  | Adjusted model | -0.154 | -0.362 | 0.055 | 0.148 | 0.096 | 0.365 |
| Receptive Language | Univariable model | -0.160 | -0.336 | 0.016 | 0.074 | 0.018 | 0.074 |
|  | Baseline model | -0.079 | -0.283 | 0.124 | 0.442 | 0.059 | 0.453 |
|  | Adjusted model | -0.072 | -0.274 | 0.129 | 0.479 | 0.156 | 0.484 |
| Expressive Language | Univariable model | **-0.203** | -0.377 | -0.028 | 0.023 | 0.033 | **0.031** |
|  | Baseline model | -0.076 | -0.274 | 0.123 | 0.453 | 0.103 | 0.453 |
|  | Adjusted model | -0.071 | -0.273 | 0.130 | 0.484 | 0.159 | 0.484 |

Baseline models are controlled for infant sex, gestational age at sampling and batch. Adjusted models additionally control for gestational age at birth, birthweight Z-score, maternal university education and SIMD.

**Supplementary Table 7.** Associations between DNAm CRP and MSEL subscales in exploratory analyses in the preterm children only at 5 years old (n = 67). Betas (standardised regression coefficients for DNAm CRP) and 95% confidence intervals are reported. Bold text indicate statistically significant associations which survive FDR correction for multiple comparisons (q <0.05).

| **MSEL subscale** | **model** | **Beta** | **Lower CI** | **Upper CI** | **p** | **R^2^** | **q** |
| --- | --- | --- | --- | --- | --- | --- | --- |
| Fine Motor | Univariable model | **-0.399** | -0.626 | -0.172 | 0.001 | 0.146 | **0.002** |
|  | Baseline model | **-0.314** | -0.568 | -0.060 | 0.016 | 0.226 | **0.033** |
|  | Adjusted model | **-0.284** | -0.531 | -0.037 | 0.025 | 0.349 | **0.050** |
| Visual Reception | Univariable model | **-0.485** | -0.701 | -0.268 | 0.000 | 0.223 | **0.000** |
|  | Baseline model | **-0.437** | -0.695 | -0.179 | 0.001 | 0.200 | **0.005** |
|  | Adjusted model | **-0.396** | -0.657 | -0.136 | 0.004 | 0.274 | **0.014** |
| Receptive Language | Univariable model | **-0.258** | -0.497 | -0.019 | 0.035 | 0.052 | **0.047** |
|  | Baseline model | -0.168 | -0.447 | 0.111 | 0.234 | 0.066 | 0.311 |
|  | Adjusted model | -0.181 | -0.458 | 0.096 | 0.196 | 0.180 | 0.261 |
| Expressive Language | Univariable model | -0.235 | -0.476 | 0.006 | 0.056 | 0.041 | 0.056 |
|  | Baseline model | -0.083 | -0.338 | 0.172 | 0.516 | 0.221 | 0.516 |
|  | Adjusted model | -0.083 | -0.347 | 0.182 | 0.533 | 0.254 | 0.533 |

Baseline models are controlled for infant sex, gestational age at sampling and batch. Adjusted models additionally control for gestational age at birth, birthweight Z-score, maternal university education and SIMD.

**Supplementary Table 8.** Williams’ test (1959) for differences in magnitudes of standardised DNAm CRP regression coefficients for baseline models between MSEL subscales: fine motor (FM), visual reception (VR), expressive language (EL), receptive language (RL). R1 indicates correlation between subscale 1 and DNAm CRP. R2 indicates correlation between subscale 2 and DNAm CRP. R_common indicates correlation between subscale 1 and subscale 2. Bold text indicate statistically significant differences (t), where p <0.05

| Subscale 1 | Subscale 2 | R1 | R2 | R_common | t | p |
| --- | --- | --- | --- | --- | --- | --- |
| FM | VR | -0.314 | -0.437 | 0.526 | 1.118 | 0.264 |
| FM | EL | -0.314 | -0.083 | 0.482 | -1.868 | 0.062 |
| FM | RL | -0.314 | -0.168 | 0.500 | -1.214 | 0.225 |
| VR | EL | -0.437 | -0.083 | 0.546 | **-3.143** | **0.002** |
| VR | RL | -0.437 | -0.168 | 0.609 | **-2.602** | **0.009** |
| EL | RL | -0.083 | -0.168 | 0.566 | 0.735 | 0.462 |

**Supplementary Table 9.** Association of EpiScores with MSEL Early Learning Composite (ELC) in the full cohort at 5 years old (n = 125). Betas (standardised regression coefficients for each EpiScore) and 95% confidence intervals are reported. Bold text indicate statistically significant associations according to the preregistered PCA-based adjusted significance threshold p <0.005. False discovery rate-adjusted p-values (q), calculated using the Benjamini-Hochberg procedure across 43 two-sided tests, are additionally provided for comparison. Baseline models are controlled for infant sex, gestational age at sampling and batch. Adjusted models additionally control for gestational age at birth, birthweight Z-score, maternal university education and SIMD.

| **EpiScore** | **model** | **Beta** | **Lower CI** | **Upper CI** | **p** | **pFDR (q)** | **R^2^** |
| --- | --- | --- | --- | --- | --- | --- | --- |
| **Afamin** | Univariable model | -0.180 | -0.355 | -0.004 | 0.045 | 0.179 | 0.024 |
|  | Baseline model | 0.028 | -0.178 | 0.234 | 0.788 | 0.842 | 0.158 |
|  | Adjusted model | 0.035 | -0.161 | 0.231 | 0.724 | 0.966 | 0.270 |
| **CCL11** | Univariable model | 0.108 | -0.069 | 0.285 | 0.231 | 0.444 | 0.004 |
|  | Baseline model | -0.078 | -0.272 | 0.116 | 0.428 | 0.602 | 0.161 |
|  | Adjusted model | -0.063 | -0.245 | 0.119 | 0.497 | 0.864 | 0.272 |
| **CCL18** | Univariable model | 0.099 | -0.078 | 0.277 | 0.270 | 0.457 | 0.002 |
|  | Baseline model | 0.088 | -0.076 | 0.253 | 0.289 | 0.509 | 0.165 |
|  | Adjusted model | 0.100 | -0.054 | 0.255 | 0.202 | 0.864 | 0.279 |
| **CCL21** | Univariable model | -0.211 | -0.386 | -0.037 | 0.018 | 0.114 | 0.037 |
|  | Baseline model | -0.151 | -0.349 | 0.048 | 0.136 | 0.398 | 0.173 |
|  | Adjusted model | -0.097 | -0.290 | 0.095 | 0.319 | 0.864 | 0.275 |
| **CCL22** | Univariable model | -0.009 | -0.187 | 0.170 | 0.922 | 0.943 | -0.008 |
|  | Baseline model | -0.097 | -0.263 | 0.069 | 0.248 | 0.476 | 0.166 |
|  | Adjusted model | -0.087 | -0.247 | 0.072 | 0.280 | 0.864 | 0.276 |
| **CCL25C.C** | Univariable model | -0.135 | -0.312 | 0.042 | 0.133 | 0.366 | 0.010 |
|  | Baseline model | 0.129 | -0.082 | 0.341 | 0.228 | 0.476 | 0.167 |
|  | Adjusted model | -0.017 | -0.237 | 0.203 | 0.877 | 0.991 | 0.269 |
| **CD163** | Univariable model | -0.077 | -0.255 | 0.101 | 0.395 | 0.561 | -0.002 |
|  | Baseline model | -0.074 | -0.238 | 0.090 | 0.372 | 0.577 | 0.163 |
|  | Adjusted model | -0.020 | -0.187 | 0.147 | 0.812 | 0.991 | 0.269 |
| **CD209_antigen** | Univariable model | **0.267** | 0.095 | 0.439 | **0.003** | 0.058 | 0.064 |
|  | Baseline model | 0.079 | -0.122 | 0.281 | 0.438 | 0.602 | 0.161 |
|  | Adjusted model | 0.002 | -0.196 | 0.200 | 0.985 | 0.994 | 0.269 |
| **CD48_antigen** | Univariable model | -0.170 | -0.346 | 0.006 | 0.058 | 0.201 | 0.021 |
|  | Baseline model | -0.143 | -0.314 | 0.028 | 0.100 | 0.339 | 0.176 |
|  | Adjusted model | -0.111 | -0.285 | 0.062 | 0.207 | 0.864 | 0.279 |
| **CD6** | Univariable model | -0.031 | -0.209 | 0.148 | 0.734 | 0.822 | -0.007 |
|  | Baseline model | -0.035 | -0.199 | 0.129 | 0.671 | 0.777 | 0.158 |
|  | Adjusted model | -0.018 | -0.176 | 0.141 | 0.826 | 0.991 | 0.269 |
| **CD5L** | Univariable model | 0.027 | -0.152 | 0.205 | 0.767 | 0.823 | -0.007 |
|  | Baseline model | -0.198 | -0.395 | 0.000 | 0.050 | 0.291 | 0.184 |
|  | Adjusted model | -0.166 | -0.361 | 0.030 | 0.096 | 0.864 | 0.286 |
| **ComplementC9** | Univariable model | -0.096 | -0.274 | 0.081 | 0.285 | 0.464 | 0.001 |
|  | Baseline model | -0.046 | -0.220 | 0.127 | 0.596 | 0.729 | 0.159 |
|  | Adjusted model | -0.045 | -0.218 | 0.128 | 0.609 | 0.864 | 0.270 |
| **Contactin.4** | Univariable model | 0.105 | -0.072 | 0.283 | 0.242 | 0.444 | 0.003 |
|  | Baseline model | 0.157 | -0.005 | 0.319 | 0.057 | 0.291 | 0.182 |
|  | Adjusted model | 0.091 | -0.075 | 0.257 | 0.281 | 0.864 | 0.276 |
| **CXCL10_Olink** | Univariable model | 0.017 | -0.162 | 0.195 | 0.855 | 0.895 | -0.008 |
|  | Baseline model | -0.072 | -0.240 | 0.095 | 0.394 | 0.577 | 0.162 |
|  | Adjusted model | -0.001 | -0.165 | 0.163 | 0.994 | 0.994 | 0.269 |
| **CXCL9** | Univariable model | 0.056 | -0.122 | 0.234 | 0.535 | 0.692 | -0.005 |
|  | Baseline model | -0.003 | -0.173 | 0.166 | 0.969 | 0.972 | 0.157 |
|  | Adjusted model | 0.086 | -0.077 | 0.250 | 0.298 | 0.864 | 0.276 |
| **CRP** | Univariable model | -0.273 | -0.445 | -0.101 | 0.002 | 0.058 | 0.067 |
|  | Baseline model | -0.165 | -0.355 | 0.025 | 0.089 | 0.339 | 0.177 |
|  | Adjusted model | -0.134 | -0.320 | 0.052 | 0.157 | 0.864 | 0.281 |
| **CRTAM** | Univariable model | -0.102 | -0.279 | 0.076 | 0.258 | 0.454 | 0.002 |
|  | Baseline model | -0.219 | -0.384 | -0.054 | 0.010 | 0.291 | 0.203 |
|  | Adjusted model | -0.123 | -0.290 | 0.045 | 0.149 | 0.864 | 0.282 |
| **FcRL2** | Univariable model | -0.065 | -0.243 | 0.113 | 0.472 | 0.648 | -0.004 |
|  | Baseline model | -0.023 | -0.189 | 0.142 | 0.779 | 0.842 | 0.158 |
|  | Adjusted model | -0.037 | -0.195 | 0.121 | 0.643 | 0.885 | 0.270 |
| **FGF.21** | Univariable model | -0.041 | -0.220 | 0.137 | 0.648 | 0.770 | -0.006 |
|  | Baseline model | -0.101 | -0.266 | 0.064 | 0.229 | 0.476 | 0.167 |
|  | Adjusted model | -0.057 | -0.217 | 0.103 | 0.483 | 0.864 | 0.272 |
| **GDF15** | Univariable model | -0.155 | -0.331 | 0.021 | 0.084 | 0.247 | 0.016 |
|  | Baseline model | -0.099 | -0.267 | 0.068 | 0.244 | 0.476 | 0.167 |
|  | Adjusted model | -0.057 | -0.217 | 0.104 | 0.485 | 0.864 | 0.272 |
| **GHR** | Univariable model | 0.029 | -0.149 | 0.208 | 0.747 | 0.822 | -0.007 |
|  | Baseline model | 0.053 | -0.110 | 0.216 | 0.522 | 0.656 | 0.160 |
|  | Adjusted model | 0.005 | -0.151 | 0.162 | 0.946 | 0.991 | 0.269 |
| **HCII** | Univariable model | 0.060 | -0.118 | 0.238 | 0.509 | 0.678 | -0.005 |
|  | Baseline model | -0.245 | -0.443 | -0.048 | 0.015 | 0.291 | 0.197 |
|  | Adjusted model | -0.186 | -0.377 | 0.004 | 0.055 | 0.864 | 0.292 |
| **HGF** | Univariable model | -0.228 | -0.401 | -0.054 | 0.011 | 0.094 | 0.044 |
|  | Baseline model | -0.159 | -0.325 | 0.006 | 0.059 | 0.291 | 0.182 |
|  | Adjusted model | -0.086 | -0.248 | 0.076 | 0.296 | 0.864 | 0.276 |
| **IGFBP.1** | Univariable model | 0.166 | -0.010 | 0.342 | 0.064 | 0.201 | 0.020 |
|  | Baseline model | -0.003 | -0.187 | 0.180 | 0.972 | 0.972 | 0.157 |
|  | Adjusted model | -0.014 | -0.186 | 0.158 | 0.870 | 0.991 | 0.269 |
| **IGFBP.4** | Univariable model | -0.091 | -0.269 | 0.087 | 0.313 | 0.492 | 0.000 |
|  | Baseline model | -0.084 | -0.247 | 0.079 | 0.310 | 0.524 | 0.164 |
|  | Adjusted model | -0.062 | -0.221 | 0.098 | 0.444 | 0.864 | 0.273 |
| **L.selectin** | Univariable model | 0.166 | -0.010 | 0.342 | 0.064 | 0.201 | 0.020 |
|  | Baseline model | 0.075 | -0.096 | 0.247 | 0.386 | 0.577 | 0.162 |
|  | Adjusted model | 0.079 | -0.084 | 0.243 | 0.339 | 0.864 | 0.275 |
| **Lactotransferrin** | Univariable model | 0.119 | -0.058 | 0.296 | 0.187 | 0.444 | 0.006 |
|  | Baseline model | 0.160 | -0.006 | 0.326 | 0.059 | 0.291 | 0.182 |
|  | Adjusted model | 0.109 | -0.056 | 0.274 | 0.192 | 0.864 | 0.280 |
| **FCGR3A** | Univariable model | -0.111 | -0.288 | 0.067 | 0.219 | 0.444 | 0.004 |
|  | Baseline model | -0.098 | -0.267 | 0.070 | 0.249 | 0.476 | 0.166 |
|  | Adjusted model | -0.047 | -0.214 | 0.120 | 0.579 | 0.864 | 0.271 |
| **MMP.9** | Univariable model | 0.046 | -0.132 | 0.225 | 0.607 | 0.742 | -0.006 |
|  | Baseline model | -0.032 | -0.202 | 0.139 | 0.715 | 0.806 | 0.158 |
|  | Adjusted model | 0.009 | -0.151 | 0.169 | 0.913 | 0.991 | 0.269 |
| **NCAM1** | Univariable model | 0.228 | 0.054 | 0.402 | 0.011 | 0.094 | 0.044 |
|  | Baseline model | 0.094 | -0.115 | 0.303 | 0.374 | 0.577 | 0.163 |
|  | Adjusted model | -0.011 | -0.218 | 0.196 | 0.916 | 0.991 | 0.269 |
| **PIGR** | Univariable model | 0.125 | -0.052 | 0.302 | 0.165 | 0.428 | 0.008 |
|  | Baseline model | -0.209 | -0.425 | 0.007 | 0.058 | 0.291 | 0.182 |
|  | Adjusted model | -0.115 | -0.324 | 0.094 | 0.279 | 0.864 | 0.276 |
| **FAP** | Univariable model | 0.000 | -0.179 | 0.178 | 0.996 | 0.996 | -0.008 |
|  | Baseline model | -0.048 | -0.235 | 0.139 | 0.615 | 0.731 | 0.159 |
|  | Adjusted model | -0.007 | -0.183 | 0.168 | 0.933 | 0.991 | 0.269 |
| **S100.A9** | Univariable model | 0.112 | -0.065 | 0.290 | 0.213 | 0.444 | 0.005 |
|  | Baseline model | 0.056 | -0.111 | 0.222 | 0.510 | 0.656 | 0.160 |
|  | Adjusted model | 0.045 | -0.115 | 0.205 | 0.576 | 0.864 | 0.271 |
| **SCGF alpha** | Univariable model | -0.047 | -0.225 | 0.131 | 0.602 | 0.742 | -0.006 |
|  | Baseline model | 0.197 | 0.015 | 0.379 | 0.034 | 0.291 | 0.188 |
|  | Adjusted model | 0.163 | -0.009 | 0.335 | 0.063 | 0.864 | 0.290 |
| **SCGF beta** | Univariable model | -0.106 | -0.284 | 0.071 | 0.239 | 0.444 | 0.003 |
|  | Baseline model | 0.196 | -0.003 | 0.395 | 0.054 | 0.291 | 0.183 |
|  | Adjusted model | 0.171 | -0.016 | 0.358 | 0.072 | 0.864 | 0.289 |
| **Semaphorin.3E** | Univariable model | 0.085 | -0.093 | 0.263 | 0.346 | 0.525 | -0.001 |
|  | Baseline model | 0.125 | -0.039 | 0.288 | 0.133 | 0.398 | 0.173 |
|  | Adjusted model | 0.063 | -0.094 | 0.220 | 0.427 | 0.864 | 0.273 |
| **SKR3** | Univariable model | 0.035 | -0.143 | 0.214 | 0.697 | 0.807 | -0.007 |
|  | Baseline model | -0.151 | -0.331 | 0.029 | 0.100 | 0.339 | 0.176 |
|  | Adjusted model | -0.081 | -0.257 | 0.096 | 0.366 | 0.864 | 0.274 |
| **SIGLEC1** | Univariable model | -0.196 | -0.371 | -0.021 | 0.029 | 0.140 | 0.030 |
|  | Baseline model | -0.145 | -0.317 | 0.028 | 0.099 | 0.339 | 0.176 |
|  | Adjusted model | -0.081 | -0.262 | 0.100 | 0.377 | 0.864 | 0.274 |
| **SLITRK5** | Univariable model | 0.242 | 0.068 | 0.415 | 0.007 | 0.094 | 0.051 |
|  | Baseline model | 0.064 | -0.122 | 0.251 | 0.497 | 0.656 | 0.160 |
|  | Adjusted model | 0.058 | -0.123 | 0.239 | 0.528 | 0.864 | 0.271 |
| **Thrombopoietin_receptor** | Univariable model | 0.205 | 0.030 | 0.379 | 0.022 | 0.122 | 0.034 |
|  | Baseline model | 0.030 | -0.205 | 0.264 | 0.803 | 0.842 | 0.157 |
|  | Adjusted model | -0.063 | -0.295 | 0.168 | 0.589 | 0.864 | 0.271 |
| **Trypsin.2** | Univariable model | -0.082 | -0.260 | 0.096 | 0.362 | 0.531 | -0.001 |
|  | Baseline model | -0.097 | -0.261 | 0.066 | 0.242 | 0.476 | 0.167 |
|  | Adjusted model | -0.012 | -0.170 | 0.147 | 0.883 | 0.991 | 0.269 |
| **VCAM1** | Univariable model | -0.115 | -0.292 | 0.063 | 0.203 | 0.444 | 0.005 |
|  | Baseline model | -0.094 | -0.261 | 0.072 | 0.264 | 0.484 | 0.166 |
|  | Adjusted model | -0.049 | -0.218 | 0.120 | 0.566 | 0.864 | 0.271 |
| **VEGFA** | Univariable model | -0.221 | -0.395 | -0.047 | 0.013 | 0.097 | 0.041 |
|  | Baseline model | -0.104 | -0.277 | 0.069 | 0.238 | 0.476 | 0.167 |
|  | Adjusted model | -0.052 | -0.218 | 0.114 | 0.536 | 0.864 | 0.271 |
| **WFIKKN2** | Univariable model | 0.192 | 0.017 | 0.367 | 0.032 | 0.141 | 0.029 |
|  | Baseline model | 0.108 | -0.058 | 0.274 | 0.199 | 0.476 | 0.169 |
|  | Adjusted model | 0.042 | -0.118 | 0.202 | 0.606 | 0.864 | 0.271 |

**Supplementary Table 10.** Association of EpiScores with Bayley-III Cognitive Composite in preterm children at 2 years old (n = 150), adjusting for number of inflammatory exposures. Betas (standardised regression coefficients for each EpiScore) and 95% confidence intervals are reported. Bold text indicate statistically significant associations according to the preregistered PCA-based adjusted significance threshold p <0.005. False discovery rate-adjusted p-values (q), calculated using the Benjamini-Hochberg procedure across 43 two-sided tests, are additionally provided for comparison. Baseline models are controlled for infant sex, gestational age at sampling and batch. Adjusted models additionally control for gestational age at birth, birthweight Z-score, maternal university education and SIMD.

| **EpiScore** | **model** | **Beta** | **Lower_CI** | **Upper_CI** | **p** | **pFDR (q)** | **adj_r2** |
| --- | --- | --- | --- | --- | --- | --- | --- |
| **Afamin** | univariable model | 0.107 | -0.055 | 0.268 | 0.193 | 0.870 | 0.005 |
| **Afamin** | baseline model | 0.202 | -0.019 | 0.422 | 0.072 | 0.637 | 0.047 |
| **Afamin** | baseline model + inflammatory exposures | 0.214 | -0.016 | 0.445 | 0.068 | 0.648 | 0.047 |
| **Afamin** | adjusted model | 0.193 | -0.030 | 0.416 | 0.090 | 0.881 | 0.085 |
| **Afamin** | adjusted model + inflammatory exposures | 0.218 | -0.014 | 0.451 | 0.066 | 0.857 | 0.099 |
| **CCL11** | univariable model | -0.060 | -0.222 | 0.102 | 0.464 | 0.870 | -0.003 |
| **CCL11** | baseline model | -0.023 | -0.212 | 0.167 | 0.812 | 0.972 | 0.025 |
| **CCL11** | baseline model + inflammatory exposures | -0.015 | -0.211 | 0.181 | 0.877 | 0.977 | 0.023 |
| **CCL11** | adjusted model | -0.012 | -0.199 | 0.175 | 0.900 | 0.943 | 0.066 |
| **CCL11** | adjusted model + inflammatory exposures | -0.012 | -0.204 | 0.179 | 0.899 | 0.988 | 0.074 |
| **CCL18** | univariable model | 0.180 | 0.020 | 0.340 | 0.028 | 0.870 | 0.026 |
| **CCL18** | baseline model | 0.198 | 0.036 | 0.361 | 0.017 | 0.637 | 0.063 |
| **CCL18** | baseline model + inflammatory exposures | 0.182 | 0.009 | 0.354 | 0.039 | 0.648 | 0.054 |
| **CCL18** | adjusted model | 0.172 | 0.006 | 0.337 | 0.043 | 0.881 | 0.093 |
| **CCL18** | adjusted model + inflammatory exposures | 0.143 | -0.030 | 0.316 | 0.105 | 0.857 | 0.093 |
| **CCL21** | univariable model | -0.137 | -0.297 | 0.024 | 0.096 | 0.870 | 0.012 |
| **CCL21** | baseline model | -0.062 | -0.261 | 0.138 | 0.543 | 0.884 | 0.027 |
| **CCL21** | baseline model + inflammatory exposures | -0.061 | -0.276 | 0.153 | 0.572 | 0.839 | 0.025 |
| **CCL21** | adjusted model | -0.030 | -0.236 | 0.176 | 0.772 | 0.915 | 0.067 |
| **CCL21** | adjusted model + inflammatory exposures | -0.029 | -0.243 | 0.186 | 0.792 | 0.988 | 0.075 |
| **CCL22** | univariable model | 0.012 | -0.150 | 0.175 | 0.881 | 0.945 | -0.007 |
| **CCL22** | baseline model | 0.055 | -0.123 | 0.234 | 0.541 | 0.884 | 0.027 |
| **CCL22** | baseline model + inflammatory exposures | 0.069 | -0.118 | 0.256 | 0.467 | 0.839 | 0.027 |
| **CCL22** | adjusted model | 0.029 | -0.148 | 0.206 | 0.746 | 0.915 | 0.067 |
| **CCL22** | adjusted model + inflammatory exposures | 0.031 | -0.153 | 0.216 | 0.737 | 0.988 | 0.075 |
| **CCL25C.C** | univariable model | 0.007 | -0.155 | 0.170 | 0.928 | 0.945 | -0.007 |
| **CCL25C.C** | baseline model | -0.048 | -0.255 | 0.160 | 0.651 | 0.884 | 0.026 |
| **CCL25C.C** | baseline model + inflammatory exposures | 0.013 | -0.203 | 0.230 | 0.904 | 0.977 | 0.023 |
| **CCL25C.C** | adjusted model | -0.073 | -0.282 | 0.136 | 0.491 | 0.915 | 0.069 |
| **CCL25C.C** | adjusted model + inflammatory exposures | -0.020 | -0.237 | 0.198 | 0.859 | 0.988 | 0.074 |
| **CD163** | univariable model | -0.019 | -0.181 | 0.144 | 0.820 | 0.945 | -0.006 |
| **CD163** | baseline model | 0.026 | -0.147 | 0.198 | 0.769 | 0.966 | 0.025 |
| **CD163** | baseline model + inflammatory exposures | 0.039 | -0.143 | 0.222 | 0.669 | 0.911 | 0.024 |
| **CD163** | adjusted model | 0.021 | -0.153 | 0.195 | 0.811 | 0.915 | 0.067 |
| **CD163** | adjusted model + inflammatory exposures | 0.043 | -0.141 | 0.227 | 0.643 | 0.988 | 0.076 |
| **CD209_antigen** | univariable model | 0.052 | -0.111 | 0.214 | 0.530 | 0.870 | -0.004 |
| **CD209_antigen** | baseline model | 0.122 | -0.104 | 0.347 | 0.287 | 0.841 | 0.032 |
| **CD209_antigen** | baseline model + inflammatory exposures | 0.126 | -0.113 | 0.365 | 0.298 | 0.820 | 0.031 |
| **CD209_antigen** | adjusted model | 0.085 | -0.141 | 0.312 | 0.458 | 0.915 | 0.070 |
| **CD209_antigen** | adjusted model + inflammatory exposures | 0.085 | -0.155 | 0.325 | 0.486 | 0.988 | 0.078 |
| **CD48_antigen** | univariable model | 0.106 | -0.056 | 0.267 | 0.197 | 0.870 | 0.005 |
| **CD48_antigen** | baseline model | 0.135 | -0.039 | 0.309 | 0.128 | 0.705 | 0.040 |
| **CD48_antigen** | baseline model + inflammatory exposures | 0.142 | -0.046 | 0.331 | 0.138 | 0.661 | 0.039 |
| **CD48_antigen** | adjusted model | 0.117 | -0.061 | 0.294 | 0.196 | 0.915 | 0.077 |
| **CD48_antigen** | adjusted model + inflammatory exposures | 0.119 | -0.073 | 0.310 | 0.222 | 0.988 | 0.085 |
| **CD6** | univariable model | 0.006 | -0.156 | 0.169 | 0.938 | 0.945 | -0.007 |
| **CD6** | baseline model | 0.002 | -0.163 | 0.167 | 0.980 | 0.996 | 0.025 |
| **CD6** | baseline model + inflammatory exposures | -0.003 | -0.174 | 0.167 | 0.969 | 0.977 | 0.023 |
| **CD6** | adjusted model | -0.022 | -0.186 | 0.143 | 0.795 | 0.915 | 0.067 |
| **CD6** | adjusted model + inflammatory exposures | -0.030 | -0.199 | 0.139 | 0.727 | 0.988 | 0.075 |
| **CD5L** | univariable model | 0.076 | -0.086 | 0.238 | 0.356 | 0.870 | -0.001 |
| **CD5L** | baseline model | 0.238 | 0.016 | 0.460 | 0.036 | 0.637 | 0.055 |
| **CD5L** | baseline model + inflammatory exposures | 0.202 | -0.033 | 0.437 | 0.092 | 0.648 | 0.044 |
| **CD5L** | adjusted model | 0.241 | 0.020 | 0.463 | 0.033 | 0.881 | 0.096 |
| **CD5L** | adjusted model + inflammatory exposures | 0.214 | -0.018 | 0.447 | 0.071 | 0.857 | 0.098 |
| **ComplementC9** | univariable model | -0.084 | -0.245 | 0.078 | 0.309 | 0.870 | 0.000 |
| **ComplementC9** | baseline model | -0.121 | -0.302 | 0.061 | 0.192 | 0.705 | 0.036 |
| **ComplementC9** | baseline model + inflammatory exposures | -0.160 | -0.350 | 0.029 | 0.096 | 0.648 | 0.043 |
| **ComplementC9** | adjusted model | -0.096 | -0.276 | 0.083 | 0.291 | 0.915 | 0.074 |
| **ComplementC9** | adjusted model + inflammatory exposures | -0.142 | -0.329 | 0.045 | 0.136 | 0.857 | 0.090 |
| **Contactin.4** | univariable model | 0.089 | -0.073 | 0.251 | 0.278 | 0.870 | 0.001 |
| **Contactin.4** | baseline model | 0.077 | -0.091 | 0.246 | 0.366 | 0.841 | 0.030 |
| **Contactin.4** | baseline model + inflammatory exposures | 0.113 | -0.060 | 0.286 | 0.200 | 0.767 | 0.035 |
| **Contactin.4** | adjusted model | 0.053 | -0.115 | 0.220 | 0.535 | 0.915 | 0.069 |
| **Contactin.4** | adjusted model + inflammatory exposures | 0.075 | -0.097 | 0.248 | 0.389 | 0.988 | 0.080 |
| **CXCL10_Olink** | univariable model | 0.083 | -0.079 | 0.245 | 0.314 | 0.870 | 0.000 |
| **CXCL10_Olink** | baseline model | 0.089 | -0.076 | 0.254 | 0.289 | 0.841 | 0.032 |
| **CXCL10_Olink** | baseline model + inflammatory exposures | 0.080 | -0.094 | 0.255 | 0.366 | 0.839 | 0.029 |
| **CXCL10_Olink** | adjusted model | 0.078 | -0.086 | 0.243 | 0.347 | 0.915 | 0.072 |
| **CXCL10_Olink** | adjusted model + inflammatory exposures | 0.092 | -0.082 | 0.266 | 0.297 | 0.988 | 0.082 |
| **CXCL9** | univariable model | 0.107 | -0.054 | 0.269 | 0.192 | 0.870 | 0.005 |
| **CXCL9** | baseline model | 0.109 | -0.052 | 0.270 | 0.182 | 0.705 | 0.037 |
| **CXCL9** | baseline model + inflammatory exposures | 0.103 | -0.065 | 0.272 | 0.227 | 0.767 | 0.034 |
| **CXCL9** | adjusted model | 0.071 | -0.091 | 0.233 | 0.388 | 0.915 | 0.071 |
| **CXCL9** | adjusted model + inflammatory exposures | 0.073 | -0.095 | 0.242 | 0.392 | 0.988 | 0.080 |
| **CRP** | univariable model | 0.014 | -0.148 | 0.177 | 0.862 | 0.945 | -0.007 |
| **CRP** | baseline model | 0.004 | -0.191 | 0.199 | 0.967 | 0.996 | 0.025 |
| **CRP** | baseline model + inflammatory exposures | -0.004 | -0.206 | 0.198 | 0.970 | 0.977 | 0.023 |
| **CRP** | adjusted model | 0.024 | -0.170 | 0.218 | 0.807 | 0.915 | 0.067 |
| **CRP** | adjusted model + inflammatory exposures | 0.011 | -0.189 | 0.210 | 0.916 | 0.988 | 0.074 |
| **CRTAM** | univariable model | -0.050 | -0.212 | 0.112 | 0.545 | 0.870 | -0.004 |
| **CRTAM** | baseline model | -0.019 | -0.200 | 0.163 | 0.839 | 0.972 | 0.025 |
| **CRTAM** | baseline model + inflammatory exposures | -0.017 | -0.204 | 0.170 | 0.858 | 0.977 | 0.023 |
| **CRTAM** | adjusted model | -0.001 | -0.179 | 0.177 | 0.991 | 0.991 | 0.066 |
| **CRTAM** | adjusted model + inflammatory exposures | 0.001 | -0.182 | 0.184 | 0.993 | 0.993 | 0.074 |
| **FcRL2** | univariable model | -0.009 | -0.172 | 0.153 | 0.912 | 0.945 | -0.007 |
| **FcRL2** | baseline model | -0.003 | -0.171 | 0.165 | 0.975 | 0.996 | 0.025 |
| **FcRL2** | baseline model + inflammatory exposures | -0.012 | -0.186 | 0.163 | 0.895 | 0.977 | 0.023 |
| **FcRL2** | adjusted model | -0.021 | -0.190 | 0.147 | 0.803 | 0.915 | 0.067 |
| **FcRL2** | adjusted model + inflammatory exposures | -0.031 | -0.207 | 0.144 | 0.726 | 0.988 | 0.075 |
| **FGF.21** | univariable model | -0.064 | -0.226 | 0.098 | 0.434 | 0.870 | -0.003 |
| **FGF.21** | baseline model | -0.059 | -0.221 | 0.102 | 0.470 | 0.862 | 0.028 |
| **FGF.21** | baseline model + inflammatory exposures | -0.093 | -0.265 | 0.079 | 0.287 | 0.820 | 0.031 |
| **FGF.21** | adjusted model | -0.016 | -0.177 | 0.145 | 0.844 | 0.928 | 0.066 |
| **FGF.21** | adjusted model + inflammatory exposures | -0.042 | -0.213 | 0.130 | 0.633 | 0.988 | 0.076 |
| **GDF15** | univariable model | -0.128 | -0.289 | 0.033 | 0.119 | 0.870 | 0.010 |
| **GDF15** | baseline model | -0.158 | -0.322 | 0.005 | 0.058 | 0.637 | 0.049 |
| **GDF15** | baseline model + inflammatory exposures | -0.165 | -0.338 | 0.008 | 0.062 | 0.648 | 0.048 |
| **GDF15** | adjusted model | -0.151 | -0.314 | 0.012 | 0.069 | 0.881 | 0.088 |
| **GDF15** | adjusted model + inflammatory exposures | -0.157 | -0.327 | 0.013 | 0.070 | 0.857 | 0.098 |
| **GHR** | univariable model | -0.060 | -0.223 | 0.102 | 0.462 | 0.870 | -0.003 |
| **GHR** | baseline model | -0.081 | -0.255 | 0.093 | 0.360 | 0.841 | 0.030 |
| **GHR** | baseline model + inflammatory exposures | -0.071 | -0.255 | 0.113 | 0.448 | 0.839 | 0.027 |
| **GHR** | adjusted model | -0.098 | -0.269 | 0.074 | 0.262 | 0.915 | 0.075 |
| **GHR** | adjusted model + inflammatory exposures | -0.087 | -0.269 | 0.094 | 0.343 | 0.988 | 0.081 |
| **HCII** | univariable model | -0.055 | -0.217 | 0.108 | 0.508 | 0.870 | -0.004 |
| **HCII** | baseline model | -0.130 | -0.320 | 0.059 | 0.176 | 0.705 | 0.037 |
| **HCII** | baseline model + inflammatory exposures | -0.146 | -0.343 | 0.051 | 0.146 | 0.661 | 0.038 |
| **HCII** | adjusted model | -0.126 | -0.317 | 0.066 | 0.197 | 0.915 | 0.077 |
| **HCII** | adjusted model + inflammatory exposures | -0.128 | -0.327 | 0.070 | 0.203 | 0.988 | 0.086 |
| **HGF** | univariable model | 0.055 | -0.107 | 0.217 | 0.502 | 0.870 | -0.004 |
| **HGF** | baseline model | 0.073 | -0.106 | 0.252 | 0.422 | 0.843 | 0.029 |
| **HGF** | baseline model + inflammatory exposures | 0.079 | -0.109 | 0.267 | 0.409 | 0.839 | 0.028 |
| **HGF** | adjusted model | 0.053 | -0.127 | 0.232 | 0.563 | 0.915 | 0.068 |
| **HGF** | adjusted model + inflammatory exposures | 0.070 | -0.117 | 0.257 | 0.460 | 0.988 | 0.078 |
| **IGFBP.1** | univariable model | 0.044 | -0.119 | 0.206 | 0.597 | 0.870 | -0.005 |
| **IGFBP.1** | baseline model | -0.063 | -0.263 | 0.137 | 0.535 | 0.884 | 0.027 |
| **IGFBP.1** | baseline model + inflammatory exposures | -0.043 | -0.252 | 0.165 | 0.683 | 0.911 | 0.024 |
| **IGFBP.1** | adjusted model | -0.051 | -0.251 | 0.150 | 0.618 | 0.915 | 0.068 |
| **IGFBP.1** | adjusted model + inflammatory exposures | -0.047 | -0.254 | 0.160 | 0.654 | 0.988 | 0.076 |
| **IGFBP.4** | univariable model | -0.081 | -0.243 | 0.081 | 0.324 | 0.870 | 0.000 |
| **IGFBP.4** | baseline model | -0.088 | -0.250 | 0.074 | 0.286 | 0.841 | 0.033 |
| **IGFBP.4** | baseline model + inflammatory exposures | -0.098 | -0.264 | 0.069 | 0.247 | 0.777 | 0.033 |
| **IGFBP.4** | adjusted model | -0.072 | -0.233 | 0.088 | 0.375 | 0.915 | 0.072 |
| **IGFBP.4** | adjusted model + inflammatory exposures | -0.083 | -0.247 | 0.082 | 0.321 | 0.988 | 0.081 |
| **L.selectin** | univariable model | 0.025 | -0.137 | 0.187 | 0.762 | 0.945 | -0.006 |
| **L.selectin** | baseline model | 0.040 | -0.141 | 0.222 | 0.663 | 0.884 | 0.026 |
| **L.selectin** | baseline model + inflammatory exposures | 0.054 | -0.133 | 0.240 | 0.569 | 0.839 | 0.025 |
| **L.selectin** | adjusted model | 0.035 | -0.146 | 0.215 | 0.706 | 0.915 | 0.067 |
| **L.selectin** | adjusted model + inflammatory exposures | 0.043 | -0.143 | 0.228 | 0.650 | 0.988 | 0.076 |
| **Lactotransferrin** | univariable model | 0.042 | -0.121 | 0.204 | 0.613 | 0.870 | -0.005 |
| **Lactotransferrin** | baseline model | 0.061 | -0.105 | 0.227 | 0.467 | 0.862 | 0.028 |
| **Lactotransferrin** | baseline model + inflammatory exposures | 0.051 | -0.124 | 0.225 | 0.567 | 0.839 | 0.025 |
| **Lactotransferrin** | adjusted model | 0.034 | -0.130 | 0.199 | 0.680 | 0.915 | 0.067 |
| **Lactotransferrin** | adjusted model + inflammatory exposures | 0.014 | -0.158 | 0.186 | 0.874 | 0.988 | 0.074 |
| **FCGR3A** | univariable model | 0.126 | -0.036 | 0.287 | 0.126 | 0.870 | 0.009 |
| **FCGR3A** | baseline model | 0.156 | -0.012 | 0.324 | 0.069 | 0.637 | 0.047 |
| **FCGR3A** | baseline model + inflammatory exposures | 0.161 | -0.017 | 0.340 | 0.076 | 0.648 | 0.046 |
| **FCGR3A** | adjusted model | 0.133 | -0.035 | 0.302 | 0.120 | 0.881 | 0.082 |
| **FCGR3A** | adjusted model + inflammatory exposures | 0.136 | -0.042 | 0.315 | 0.134 | 0.857 | 0.091 |
| **MMP.9** | univariable model | -0.006 | -0.168 | 0.157 | 0.945 | 0.945 | -0.007 |
| **MMP.9** | baseline model | -0.001 | -0.251 | 0.250 | 0.996 | 0.996 | 0.025 |
| **MMP.9** | baseline model + inflammatory exposures | 0.016 | -0.242 | 0.274 | 0.901 | 0.977 | 0.023 |
| **MMP.9** | adjusted model | -0.031 | -0.279 | 0.218 | 0.808 | 0.915 | 0.067 |
| **MMP.9** | adjusted model + inflammatory exposures | -0.006 | -0.259 | 0.248 | 0.965 | 0.988 | 0.074 |
| **NCAM1** | univariable model | 0.021 | -0.142 | 0.183 | 0.802 | 0.945 | -0.006 |
| **NCAM1** | baseline model | 0.119 | -0.119 | 0.358 | 0.325 | 0.841 | 0.031 |
| **NCAM1** | baseline model + inflammatory exposures | 0.107 | -0.138 | 0.353 | 0.389 | 0.839 | 0.028 |
| **NCAM1** | adjusted model | 0.035 | -0.207 | 0.277 | 0.777 | 0.915 | 0.067 |
| **NCAM1** | adjusted model + inflammatory exposures | 0.012 | -0.236 | 0.260 | 0.922 | 0.988 | 0.074 |
| **PIGR** | univariable model | -0.082 | -0.244 | 0.080 | 0.317 | 0.870 | 0.000 |
| **PIGR** | baseline model | -0.090 | -0.308 | 0.127 | 0.414 | 0.843 | 0.029 |
| **PIGR** | baseline model + inflammatory exposures | -0.101 | -0.326 | 0.125 | 0.379 | 0.839 | 0.028 |
| **PIGR** | adjusted model | -0.090 | -0.305 | 0.125 | 0.407 | 0.915 | 0.071 |
| **PIGR** | adjusted model + inflammatory exposures | -0.089 | -0.309 | 0.132 | 0.428 | 0.988 | 0.079 |
| **FAP** | univariable model | -0.014 | -0.177 | 0.148 | 0.863 | 0.945 | -0.007 |
| **FAP** | baseline model | 0.040 | -0.137 | 0.216 | 0.656 | 0.884 | 0.026 |
| **FAP** | baseline model + inflammatory exposures | 0.011 | -0.179 | 0.202 | 0.907 | 0.977 | 0.023 |
| **FAP** | adjusted model | 0.031 | -0.144 | 0.206 | 0.724 | 0.915 | 0.067 |
| **FAP** | adjusted model + inflammatory exposures | -0.005 | -0.190 | 0.181 | 0.960 | 0.988 | 0.074 |
| **S100.A9** | univariable model | 0.009 | -0.154 | 0.171 | 0.915 | 0.945 | -0.007 |
| **S100.A9** | baseline model | -0.003 | -0.168 | 0.162 | 0.972 | 0.996 | 0.025 |
| **S100.A9** | baseline model + inflammatory exposures | 0.002 | -0.168 | 0.173 | 0.977 | 0.977 | 0.023 |
| **S100.A9** | adjusted model | -0.028 | -0.195 | 0.138 | 0.739 | 0.915 | 0.067 |
| **S100.A9** | adjusted model + inflammatory exposures | -0.017 | -0.189 | 0.155 | 0.847 | 0.988 | 0.075 |
| **SCGF_alpha** | univariable model | 0.098 | -0.064 | 0.260 | 0.233 | 0.870 | 0.003 |
| **SCGF_alpha** | baseline model | 0.157 | -0.038 | 0.352 | 0.114 | 0.705 | 0.042 |
| **SCGF_alpha** | baseline model + inflammatory exposures | 0.150 | -0.055 | 0.354 | 0.150 | 0.661 | 0.038 |
| **SCGF_alpha** | adjusted model | 0.100 | -0.100 | 0.301 | 0.325 | 0.915 | 0.073 |
| **SCGF_alpha** | adjusted model + inflammatory exposures | 0.085 | -0.123 | 0.293 | 0.421 | 0.988 | 0.079 |
| **SCGF_beta** | univariable model | 0.070 | -0.092 | 0.233 | 0.391 | 0.870 | -0.002 |
| **SCGF_beta** | baseline model | 0.138 | -0.067 | 0.342 | 0.185 | 0.705 | 0.037 |
| **SCGF_beta** | baseline model + inflammatory exposures | 0.135 | -0.082 | 0.352 | 0.220 | 0.767 | 0.034 |
| **SCGF_beta** | adjusted model | 0.080 | -0.130 | 0.289 | 0.455 | 0.915 | 0.070 |
| **SCGF_beta** | adjusted model + inflammatory exposures | 0.068 | -0.151 | 0.287 | 0.543 | 0.988 | 0.077 |
| **Semaphorin.3E** | univariable model | 0.012 | -0.150 | 0.175 | 0.880 | 0.945 | -0.007 |
| **Semaphorin.3E** | baseline model | 0.015 | -0.208 | 0.238 | 0.893 | 0.996 | 0.025 |
| **Semaphorin.3E** | baseline model + inflammatory exposures | 0.009 | -0.221 | 0.238 | 0.942 | 0.977 | 0.023 |
| **Semaphorin.3E** | adjusted model | 0.001 | -0.219 | 0.222 | 0.989 | 0.991 | 0.066 |
| **Semaphorin.3E** | adjusted model + inflammatory exposures | -0.019 | -0.244 | 0.207 | 0.869 | 0.988 | 0.074 |
| **SKR3** | univariable model | 0.045 | -0.118 | 0.207 | 0.587 | 0.870 | -0.005 |
| **SKR3** | baseline model | 0.044 | -0.134 | 0.221 | 0.626 | 0.884 | 0.026 |
| **SKR3** | baseline model + inflammatory exposures | 0.069 | -0.114 | 0.253 | 0.458 | 0.839 | 0.027 |
| **SKR3** | adjusted model | 0.038 | -0.141 | 0.216 | 0.678 | 0.915 | 0.067 |
| **SKR3** | adjusted model + inflammatory exposures | 0.081 | -0.105 | 0.267 | 0.390 | 0.988 | 0.080 |
| **SIGLEC1** | univariable model | 0.051 | -0.112 | 0.213 | 0.538 | 0.870 | -0.004 |
| **SIGLEC1** | baseline model | 0.083 | -0.104 | 0.269 | 0.382 | 0.841 | 0.030 |
| **SIGLEC1** | baseline model + inflammatory exposures | 0.067 | -0.129 | 0.263 | 0.503 | 0.839 | 0.026 |
| **SIGLEC1** | adjusted model | 0.060 | -0.130 | 0.250 | 0.531 | 0.915 | 0.069 |
| **SIGLEC1** | adjusted model + inflammatory exposures | 0.058 | -0.142 | 0.257 | 0.568 | 0.988 | 0.077 |
| **SLITRK5** | univariable model | 0.047 | -0.116 | 0.209 | 0.571 | 0.870 | -0.005 |
| **SLITRK5** | baseline model | 0.057 | -0.184 | 0.298 | 0.640 | 0.884 | 0.026 |
| **SLITRK5** | baseline model + inflammatory exposures | 0.082 | -0.167 | 0.330 | 0.516 | 0.839 | 0.026 |
| **SLITRK5** | adjusted model | 0.041 | -0.197 | 0.279 | 0.732 | 0.915 | 0.067 |
| **SLITRK5** | adjusted model + inflammatory exposures | 0.057 | -0.189 | 0.303 | 0.646 | 0.988 | 0.076 |
| **Thrombopoietin_receptor** | univariable model | -0.071 | -0.233 | 0.092 | 0.391 | 0.870 | -0.002 |
| **Thrombopoietin_receptor** | baseline model | -0.046 | -0.284 | 0.192 | 0.702 | 0.908 | 0.026 |
| **Thrombopoietin_receptor** | baseline model + inflammatory exposures | -0.017 | -0.264 | 0.229 | 0.889 | 0.977 | 0.023 |
| **Thrombopoietin_receptor** | adjusted model | -0.055 | -0.292 | 0.183 | 0.649 | 0.915 | 0.068 |
| **Thrombopoietin_receptor** | adjusted model + inflammatory exposures | -0.036 | -0.281 | 0.209 | 0.773 | 0.988 | 0.075 |
| **Trypsin.2** | univariable model | -0.023 | -0.186 | 0.139 | 0.775 | 0.945 | -0.006 |
| **Trypsin.2** | baseline model | -0.018 | -0.183 | 0.146 | 0.826 | 0.972 | 0.025 |
| **Trypsin.2** | baseline model + inflammatory exposures | -0.057 | -0.228 | 0.115 | 0.516 | 0.839 | 0.026 |
| **Trypsin.2** | adjusted model | 0.013 | -0.150 | 0.176 | 0.875 | 0.939 | 0.066 |
| **Trypsin.2** | adjusted model + inflammatory exposures | -0.012 | -0.182 | 0.158 | 0.889 | 0.988 | 0.074 |
| **VCAM1** | univariable model | 0.090 | -0.072 | 0.252 | 0.274 | 0.870 | 0.001 |
| **VCAM1** | baseline model | 0.147 | -0.024 | 0.318 | 0.091 | 0.667 | 0.044 |
| **VCAM1** | baseline model + inflammatory exposures | 0.150 | -0.031 | 0.330 | 0.103 | 0.648 | 0.042 |
| **VCAM1** | adjusted model | 0.144 | -0.029 | 0.317 | 0.101 | 0.881 | 0.084 |
| **VCAM1** | adjusted model + inflammatory exposures | 0.155 | -0.028 | 0.337 | 0.096 | 0.857 | 0.094 |
| **VEGFA** | univariable model | 0.042 | -0.120 | 0.204 | 0.611 | 0.870 | -0.005 |
| **VEGFA** | baseline model | 0.052 | -0.134 | 0.239 | 0.580 | 0.884 | 0.027 |
| **VEGFA** | baseline model + inflammatory exposures | 0.049 | -0.146 | 0.243 | 0.620 | 0.880 | 0.025 |
| **VEGFA** | adjusted model | 0.033 | -0.153 | 0.220 | 0.726 | 0.915 | 0.067 |
| **VEGFA** | adjusted model + inflammatory exposures | 0.063 | -0.132 | 0.257 | 0.526 | 0.988 | 0.077 |
| **WFIKKN2** | univariable model | 0.104 | -0.057 | 0.266 | 0.205 | 0.870 | 0.004 |
| **WFIKKN2** | baseline model | 0.097 | -0.091 | 0.284 | 0.310 | 0.841 | 0.032 |
| **WFIKKN2** | baseline model + inflammatory exposures | 0.096 | -0.099 | 0.292 | 0.330 | 0.839 | 0.030 |
| **WFIKKN2** | adjusted model | 0.051 | -0.138 | 0.239 | 0.594 | 0.915 | 0.068 |
| **WFIKKN2** | adjusted model + inflammatory exposures | 0.060 | -0.135 | 0.256 | 0.541 | 0.988 | 0.077 |
|  |  |  |  |  |  |  |  |

**Supplementary Table 11.** Associations between EpiScores and MSEL Early Learning Composite in preterm children only at 5 years old, adjusting for number of inflammatory exposures. Betas (standardised regression coefficients for each EpiScore) and 95% confidence intervals are reported. Bold text indicate statistically significant associations according to the preregistered PCA-based adjusted significance threshold p <0.005. False discovery rate-adjusted p-values (q), calculated using the Benjamini-Hochberg procedure across 43 two-sided tests, are additionally provided for comparison. Baseline models are controlled for infant sex, gestational age at sampling and batch. Adjusted models additionally control for gestational age at birth, birthweight Z-score, maternal university education and SIMD.

| **EpiScore** | **model** | **Beta** | **Lower_CI** | **Upper_CI** | **pval** | **pFDR (q)** | **adj_r2** |
| --- | --- | --- | --- | --- | --- | --- | --- |
| **Afamin** | univariable model | -0.165 | -0.409 | 0.080 | 0.183 | 0.336 | 0.012 |
| **Afamin** | baseline model | 0.209 | -0.078 | 0.495 | 0.151 | 0.561 | 0.217 |
| **Afamin** | baseline model + inflammatory exposures | 0.205 | -0.088 | 0.498 | 0.167 | 0.606 | 0.219 |
| **Afamin** | adjusted model | 0.129 | -0.140 | 0.397 | 0.341 | 0.873 | 0.337 |
| **Afamin** | adjusted model + inflammatory exposures | 0.117 | -0.159 | 0.393 | 0.398 | 0.850 | 0.334 |
| **CCL11** | univariable model | 0.408 | 0.182 | 0.634 | **0.001** | 0.009 | 0.154 |
| **CCL11** | baseline model | 0.241 | -0.028 | 0.509 | 0.078 | 0.483 | 0.230 |
| **CCL11** | baseline model + inflammatory exposures | 0.232 | -0.043 | 0.506 | 0.096 | 0.590 | 0.231 |
| **CCL11** | adjusted model | 0.236 | -0.009 | 0.482 | 0.059 | 0.650 | 0.367 |
| **CCL11** | adjusted model + inflammatory exposures | 0.239 | -0.012 | 0.489 | 0.062 | 0.681 | 0.368 |
| **CCL18** | univariable model | 0.105 | -0.141 | 0.352 | 0.397 | 0.602 | -0.004 |
| **CCL18** | baseline model | 0.093 | -0.129 | 0.316 | 0.406 | 0.690 | 0.199 |
| **CCL18** | baseline model + inflammatory exposures | 0.129 | -0.108 | 0.367 | 0.281 | 0.606 | 0.208 |
| **CCL18** | adjusted model | 0.163 | -0.042 | 0.368 | 0.118 | 0.865 | 0.355 |
| **CCL18** | adjusted model + inflammatory exposures | 0.163 | -0.055 | 0.381 | 0.140 | 0.850 | 0.352 |
| **CCL21** | univariable model | -0.287 | -0.524 | -0.050 | 0.019 | 0.136 | 0.068 |
| **CCL21** | baseline model | -0.200 | -0.486 | 0.087 | 0.169 | 0.571 | 0.215 |
| **CCL21** | baseline model + inflammatory exposures | -0.233 | -0.539 | 0.074 | 0.134 | 0.590 | 0.223 |
| **CCL21** | adjusted model | -0.138 | -0.405 | 0.130 | 0.308 | 0.873 | 0.339 |
| **CCL21** | adjusted model + inflammatory exposures | -0.197 | -0.479 | 0.085 | 0.168 | 0.850 | 0.349 |
| **CCL22** | univariable model | 0.081 | -0.166 | 0.328 | 0.513 | 0.645 | -0.009 |
| **CCL22** | baseline model | 0.028 | -0.197 | 0.252 | 0.807 | 0.987 | 0.191 |
| **CCL22** | baseline model + inflammatory exposures | 0.029 | -0.207 | 0.264 | 0.809 | 0.914 | 0.193 |
| **CCL22** | adjusted model | 0.023 | -0.185 | 0.232 | 0.823 | 0.929 | 0.327 |
| **CCL22** | adjusted model + inflammatory exposures | 0.004 | -0.213 | 0.222 | 0.968 | 0.968 | 0.325 |
| **CCL25C.C** | univariable model | -0.185 | -0.428 | 0.059 | 0.135 | 0.310 | 0.019 |
| **CCL25C.C** | baseline model | 0.170 | -0.130 | 0.470 | 0.262 | 0.607 | 0.207 |
| **CCL25C.C** | baseline model + inflammatory exposures | 0.158 | -0.157 | 0.473 | 0.320 | 0.606 | 0.206 |
| **CCL25C.C** | adjusted model | 0.026 | -0.261 | 0.313 | 0.855 | 0.929 | 0.327 |
| **CCL25C.C** | adjusted model + inflammatory exposures | 0.025 | -0.275 | 0.325 | 0.868 | 0.931 | 0.325 |
| **CD163** | univariable model | -0.092 | -0.339 | 0.155 | 0.459 | 0.632 | -0.007 |
| **CD163** | baseline model | 0.002 | -0.231 | 0.235 | 0.986 | 1.000 | 0.190 |
| **CD163** | baseline model + inflammatory exposures | -0.041 | -0.290 | 0.207 | 0.742 | 0.907 | 0.194 |
| **CD163** | adjusted model | 0.015 | -0.206 | 0.235 | 0.894 | 0.929 | 0.327 |
| **CD163** | adjusted model + inflammatory exposures | -0.012 | -0.251 | 0.226 | 0.918 | 0.939 | 0.325 |
| **CD209_antigen** | univariable model | 0.335 | 0.101 | 0.568 | 0.006 | 0.062 | 0.098 |
| **CD209_antigen** | baseline model | 0.096 | -0.180 | 0.373 | 0.489 | 0.718 | 0.197 |
| **CD209_antigen** | baseline model + inflammatory exposures | 0.130 | -0.156 | 0.415 | 0.366 | 0.606 | 0.204 |
| **CD209_antigen** | adjusted model | 0.098 | -0.160 | 0.357 | 0.450 | 0.900 | 0.333 |
| **CD209_antigen** | adjusted model + inflammatory exposures | 0.130 | -0.136 | 0.396 | 0.330 | 0.850 | 0.337 |
| **CD48_antigen** | univariable model | -0.201 | -0.443 | 0.042 | 0.104 | 0.292 | 0.025 |
| **CD48_antigen** | baseline model | -0.075 | -0.328 | 0.178 | 0.558 | 0.754 | 0.195 |
| **CD48_antigen** | baseline model + inflammatory exposures | -0.109 | -0.376 | 0.158 | 0.418 | 0.613 | 0.201 |
| **CD48_antigen** | adjusted model | -0.099 | -0.338 | 0.141 | 0.413 | 0.873 | 0.334 |
| **CD48_antigen** | adjusted model + inflammatory exposures | -0.133 | -0.388 | 0.123 | 0.303 | 0.850 | 0.339 |
| **CD6** | univariable model | 0.084 | -0.163 | 0.331 | 0.499 | 0.645 | -0.008 |
| **CD6** | baseline model | 0.186 | -0.042 | 0.413 | 0.108 | 0.526 | 0.224 |
| **CD6** | baseline model + inflammatory exposures | 0.192 | -0.044 | 0.428 | 0.109 | 0.590 | 0.228 |
| **CD6** | adjusted model | 0.097 | -0.119 | 0.314 | 0.372 | 0.873 | 0.336 |
| **CD6** | adjusted model + inflammatory exposures | 0.103 | -0.124 | 0.330 | 0.365 | 0.850 | 0.335 |
| **CD5L** | univariable model | 0.182 | -0.062 | 0.425 | 0.141 | 0.310 | 0.018 |
| **CD5L** | baseline model | -0.002 | -0.259 | 0.255 | 0.988 | 1.000 | 0.190 |
| **CD5L** | baseline model + inflammatory exposures | -0.032 | -0.297 | 0.233 | 0.810 | 0.914 | 0.193 |
| **CD5L** | adjusted model | 0.024 | -0.218 | 0.267 | 0.840 | 0.929 | 0.327 |
| **CD5L** | adjusted model + inflammatory exposures | 0.028 | -0.228 | 0.285 | 0.825 | 0.931 | 0.326 |
| **ComplementC9** | univariable model | -0.240 | -0.481 | 0.000 | 0.050 | 0.191 | 0.043 |
| **ComplementC9** | baseline model | -0.152 | -0.396 | 0.092 | 0.217 | 0.607 | 0.210 |
| **ComplementC9** | baseline model + inflammatory exposures | -0.139 | -0.396 | 0.118 | 0.283 | 0.606 | 0.208 |
| **ComplementC9** | adjusted model | -0.154 | -0.393 | 0.085 | 0.202 | 0.873 | 0.345 |
| **ComplementC9** | adjusted model + inflammatory exposures | -0.124 | -0.386 | 0.138 | 0.348 | 0.850 | 0.336 |
| **Contactin.4** | univariable model | 0.083 | -0.163 | 0.330 | 0.502 | 0.645 | -0.008 |
| **Contactin.4** | baseline model | 0.104 | -0.125 | 0.333 | 0.369 | 0.690 | 0.201 |
| **Contactin.4** | baseline model + inflammatory exposures | 0.122 | -0.121 | 0.365 | 0.318 | 0.606 | 0.206 |
| **Contactin.4** | adjusted model | 0.095 | -0.123 | 0.314 | 0.386 | 0.873 | 0.335 |
| **Contactin.4** | adjusted model + inflammatory exposures | 0.131 | -0.096 | 0.359 | 0.252 | 0.850 | 0.342 |
| **CXCL10_Olink** | univariable model | 0.015 | -0.232 | 0.263 | 0.901 | 0.915 | -0.015 |
| **CXCL10_Olink** | baseline model | -0.097 | -0.326 | 0.131 | 0.397 | 0.690 | 0.200 |
| **CXCL10_Olink** | baseline model + inflammatory exposures | -0.130 | -0.368 | 0.108 | 0.278 | 0.606 | 0.209 |
| **CXCL10_Olink** | adjusted model | 0.008 | -0.213 | 0.230 | 0.939 | 0.939 | 0.327 |
| **CXCL10_Olink** | adjusted model + inflammatory exposures | -0.013 | -0.249 | 0.224 | 0.915 | 0.939 | 0.325 |
| **CXCL9** | univariable model | -0.055 | -0.302 | 0.192 | 0.659 | 0.724 | -0.012 |
| **CXCL9** | baseline model | -0.105 | -0.336 | 0.125 | 0.365 | 0.690 | 0.201 |
| **CXCL9** | baseline model + inflammatory exposures | -0.110 | -0.349 | 0.129 | 0.360 | 0.606 | 0.204 |
| **CXCL9** | adjusted model | -0.027 | -0.246 | 0.191 | 0.804 | 0.929 | 0.327 |
| **CXCL9** | adjusted model + inflammatory exposures | -0.041 | -0.266 | 0.184 | 0.717 | 0.931 | 0.327 |
| **CRP** | univariable model | -0.427 | -0.651 | -0.203 | **0.000** | 0.009 | 0.009 |
| **CRP** | baseline model | -0.307 | -0.555 | -0.059 | 0.016 | 0.355 | 0.355 |
| **CRP** | baseline model + inflammatory exposures | -0.318 | -0.588 | -0.049 | 0.021 | 0.471 | 0.471 |
| **CRP** | adjusted model | -0.290 | -0.530 | -0.051 | 0.018 | 0.421 | 0.421 |
| **CRP** | adjusted model + inflammatory exposures | -0.323 | -0.573 | -0.072 | 0.013 | 0.344 | 0.344 |
| **CRTAM** | univariable model | -0.239 | -0.479 | 0.002 | 0.052 | 0.191 | 0.043 |
| **CRTAM** | baseline model | -0.307 | -0.523 | -0.090 | 0.006 | 0.273 | 0.283 |
| **CRTAM** | baseline model + inflammatory exposures | -0.307 | -0.531 | -0.083 | 0.008 | 0.354 | 0.286 |
| **CRTAM** | adjusted model | -0.247 | -0.454 | -0.040 | 0.020 | 0.421 | 0.387 |
| **CRTAM** | adjusted model + inflammatory exposures | -0.265 | -0.477 | -0.052 | 0.016 | 0.344 | 0.396 |
| **FcRL2** | univariable model | 0.064 | -0.183 | 0.311 | 0.606 | 0.711 | -0.011 |
| **FcRL2** | baseline model | 0.195 | -0.030 | 0.420 | 0.088 | 0.483 | 0.228 |
| **FcRL2** | baseline model + inflammatory exposures | 0.180 | -0.053 | 0.413 | 0.127 | 0.590 | 0.225 |
| **FcRL2** | adjusted model | 0.096 | -0.122 | 0.315 | 0.381 | 0.873 | 0.335 |
| **FcRL2** | adjusted model + inflammatory exposures | 0.103 | -0.123 | 0.329 | 0.364 | 0.850 | 0.336 |
| **FGF.21** | univariable model | -0.059 | -0.306 | 0.188 | 0.636 | 0.717 | -0.012 |
| **FGF.21** | baseline model | -0.087 | -0.314 | 0.141 | 0.450 | 0.707 | 0.198 |
| **FGF.21** | baseline model + inflammatory exposures | -0.106 | -0.341 | 0.130 | 0.372 | 0.606 | 0.203 |
| **FGF.21** | adjusted model | -0.069 | -0.286 | 0.148 | 0.527 | 0.929 | 0.331 |
| **FGF.21** | adjusted model + inflammatory exposures | -0.076 | -0.302 | 0.150 | 0.502 | 0.850 | 0.331 |
| **GDF15** | univariable model | -0.174 | -0.417 | 0.070 | 0.160 | 0.320 | 0.015 |
| **GDF15** | baseline model | -0.218 | -0.438 | 0.001 | 0.051 | 0.452 | 0.239 |
| **GDF15** | baseline model + inflammatory exposures | -0.211 | -0.439 | 0.016 | 0.068 | 0.590 | 0.238 |
| **GDF15** | adjusted model | -0.177 | -0.382 | 0.028 | 0.090 | 0.792 | 0.359 |
| **GDF15** | adjusted model + inflammatory exposures | -0.163 | -0.378 | 0.051 | 0.133 | 0.850 | 0.353 |
| **GHR** | univariable model | 0.176 | -0.067 | 0.420 | 0.153 | 0.320 | 0.016 |
| **GHR** | baseline model | 0.168 | -0.053 | 0.389 | 0.133 | 0.561 | 0.220 |
| **GHR** | baseline model + inflammatory exposures | 0.137 | -0.101 | 0.375 | 0.253 | 0.606 | 0.210 |
| **GHR** | adjusted model | 0.042 | -0.179 | 0.264 | 0.704 | 0.929 | 0.328 |
| **GHR** | adjusted model + inflammatory exposures | 0.023 | -0.214 | 0.260 | 0.844 | 0.931 | 0.326 |
| **HCII** | univariable model | 0.115 | -0.131 | 0.361 | 0.353 | 0.574 | -0.002 |
| **HCII** | baseline model | -0.273 | -0.544 | -0.002 | 0.048 | 0.452 | 0.240 |
| **HCII** | baseline model + inflammatory exposures | -0.265 | -0.546 | 0.016 | 0.064 | 0.590 | 0.239 |
| **HCII** | adjusted model | -0.133 | -0.405 | 0.139 | 0.332 | 0.873 | 0.337 |
| **HCII** | adjusted model + inflammatory exposures | -0.135 | -0.416 | 0.145 | 0.339 | 0.850 | 0.337 |
| **HGF** | univariable model | -0.185 | -0.429 | 0.058 | 0.133 | 0.310 | 0.020 |
| **HGF** | baseline model | -0.102 | -0.328 | 0.124 | 0.373 | 0.690 | 0.201 |
| **HGF** | baseline model + inflammatory exposures | -0.128 | -0.360 | 0.104 | 0.273 | 0.606 | 0.209 |
| **HGF** | adjusted model | -0.056 | -0.267 | 0.155 | 0.599 | 0.929 | 0.330 |
| **HGF** | adjusted model + inflammatory exposures | -0.078 | -0.298 | 0.142 | 0.481 | 0.850 | 0.331 |
| **IGFBP.1** | univariable model | 0.224 | -0.018 | 0.465 | 0.069 | 0.234 | 0.035 |
| **IGFBP.1** | baseline model | 0.003 | -0.249 | 0.255 | 0.979 | 1.000 | 0.190 |
| **IGFBP.1** | baseline model + inflammatory exposures | 0.018 | -0.248 | 0.285 | 0.891 | 0.922 | 0.192 |
| **IGFBP.1** | adjusted model | 0.030 | -0.201 | 0.261 | 0.793 | 0.929 | 0.327 |
| **IGFBP.1** | adjusted model + inflammatory exposures | 0.060 | -0.184 | 0.305 | 0.622 | 0.931 | 0.328 |
| **IGFBP.4** | univariable model | -0.096 | -0.342 | 0.151 | 0.440 | 0.625 | -0.006 |
| **IGFBP.4** | baseline model | -0.024 | -0.252 | 0.205 | 0.837 | 0.995 | 0.191 |
| **IGFBP.4** | baseline model + inflammatory exposures | -0.016 | -0.249 | 0.218 | 0.894 | 0.922 | 0.192 |
| **IGFBP.4** | adjusted model | -0.093 | -0.308 | 0.122 | 0.390 | 0.873 | 0.335 |
| **IGFBP.4** | adjusted model + inflammatory exposures | -0.083 | -0.304 | 0.138 | 0.452 | 0.850 | 0.332 |
| **L.selectin** | univariable model | 0.151 | -0.094 | 0.396 | 0.222 | 0.391 | 0.008 |
| **L.selectin** | baseline model | -0.035 | -0.280 | 0.209 | 0.773 | 0.971 | 0.191 |
| **L.selectin** | baseline model + inflammatory exposures | -0.018 | -0.270 | 0.233 | 0.884 | 0.922 | 0.192 |
| **L.selectin** | adjusted model | 0.032 | -0.198 | 0.262 | 0.783 | 0.929 | 0.327 |
| **L.selectin** | adjusted model + inflammatory exposures | 0.038 | -0.199 | 0.274 | 0.752 | 0.931 | 0.326 |
| **Lactotransferrin** | univariable model | 0.022 | -0.226 | 0.269 | 0.862 | 0.903 | -0.015 |
| **Lactotransferrin** | baseline model | -0.011 | -0.258 | 0.237 | 0.931 | 1.000 | 0.190 |
| **Lactotransferrin** | baseline model + inflammatory exposures | 0.002 | -0.259 | 0.262 | 0.990 | 0.990 | 0.192 |
| **Lactotransferrin** | adjusted model | -0.053 | -0.282 | 0.175 | 0.643 | 0.929 | 0.329 |
| **Lactotransferrin** | adjusted model + inflammatory exposures | -0.041 | -0.282 | 0.199 | 0.732 | 0.931 | 0.327 |
| **FCGR3A** | univariable model | -0.165 | -0.409 | 0.080 | 0.183 | 0.336 | 0.012 |
| **FCGR3A** | baseline model | -0.068 | -0.304 | 0.168 | 0.565 | 0.754 | 0.195 |
| **FCGR3A** | baseline model + inflammatory exposures | -0.089 | -0.334 | 0.157 | 0.474 | 0.651 | 0.199 |
| **FCGR3A** | adjusted model | -0.063 | -0.285 | 0.160 | 0.576 | 0.929 | 0.330 |
| **FCGR3A** | adjusted model + inflammatory exposures | -0.082 | -0.316 | 0.152 | 0.486 | 0.850 | 0.331 |
| **MMP.9** | univariable model | 0.013 | -0.234 | 0.261 | 0.915 | 0.915 | -0.015 |
| **MMP.9** | baseline model | -0.146 | -0.377 | 0.085 | 0.210 | 0.607 | 0.211 |
| **MMP.9** | baseline model + inflammatory exposures | -0.138 | -0.376 | 0.101 | 0.253 | 0.606 | 0.210 |
| **MMP.9** | adjusted model | -0.093 | -0.310 | 0.124 | 0.393 | 0.873 | 0.335 |
| **MMP.9** | adjusted model + inflammatory exposures | -0.102 | -0.326 | 0.123 | 0.368 | 0.850 | 0.335 |
| **NCAM1** | univariable model | 0.418 | 0.193 | 0.643 | **0.000** | 0.009 | 0.162 |
| **NCAM1** | baseline model | 0.294 | 0.013 | 0.574 | 0.040 | 0.452 | 0.244 |
| **NCAM1** | baseline model + inflammatory exposures | 0.274 | -0.020 | 0.568 | 0.067 | 0.590 | 0.238 |
| **NCAM1** | adjusted model | 0.296 | 0.032 | 0.560 | 0.029 | 0.421 | 0.380 |
| **NCAM1** | adjusted model + inflammatory exposures | 0.303 | 0.027 | 0.578 | 0.032 | 0.465 | 0.382 |
| **PIGR** | univariable model | 0.238 | -0.002 | 0.479 | 0.052 | 0.191 | 0.042 |
| **PIGR** | baseline model | -0.130 | -0.443 | 0.182 | 0.408 | 0.690 | 0.199 |
| **PIGR** | baseline model + inflammatory exposures | -0.135 | -0.456 | 0.187 | 0.405 | 0.613 | 0.202 |
| **PIGR** | adjusted model | -0.087 | -0.376 | 0.202 | 0.550 | 0.929 | 0.331 |
| **PIGR** | adjusted model + inflammatory exposures | -0.104 | -0.402 | 0.195 | 0.488 | 0.850 | 0.331 |
| **FAP** | univariable model | 0.096 | -0.151 | 0.343 | 0.439 | 0.625 | -0.006 |
| **FAP** | baseline model | -0.012 | -0.253 | 0.229 | 0.921 | 1.000 | 0.190 |
| **FAP** | baseline model + inflammatory exposures | 0.070 | -0.191 | 0.331 | 0.595 | 0.793 | 0.196 |
| **FAP** | adjusted model | 0.022 | -0.201 | 0.245 | 0.843 | 0.929 | 0.327 |
| **FAP** | adjusted model + inflammatory exposures | 0.088 | -0.153 | 0.329 | 0.469 | 0.850 | 0.332 |
| **S100.A9** | univariable model | -0.041 | -0.288 | 0.207 | 0.744 | 0.798 | -0.014 |
| **S100.A9** | baseline model | -0.217 | -0.448 | 0.014 | 0.065 | 0.476 | 0.234 |
| **S100.A9** | baseline model + inflammatory exposures | -0.210 | -0.447 | 0.027 | 0.081 | 0.590 | 0.234 |
| **S100.A9** | adjusted model | -0.121 | -0.348 | 0.106 | 0.291 | 0.873 | 0.339 |
| **S100.A9** | adjusted model + inflammatory exposures | -0.124 | -0.358 | 0.110 | 0.293 | 0.850 | 0.339 |
| **SCGF_alpha** | univariable model | -0.124 | -0.370 | 0.122 | 0.318 | 0.538 | 0.000 |
| **SCGF_alpha** | baseline model | 0.115 | -0.143 | 0.373 | 0.376 | 0.690 | 0.201 |
| **SCGF_alpha** | baseline model + inflammatory exposures | 0.117 | -0.160 | 0.395 | 0.401 | 0.613 | 0.202 |
| **SCGF_alpha** | adjusted model | 0.075 | -0.172 | 0.322 | 0.547 | 0.929 | 0.331 |
| **SCGF_alpha** | adjusted model + inflammatory exposures | 0.046 | -0.226 | 0.318 | 0.737 | 0.931 | 0.326 |
| **SCGF_beta** | univariable model | -0.186 | -0.429 | 0.058 | 0.132 | 0.310 | 0.020 |
| **SCGF_beta** | baseline model | 0.165 | -0.120 | 0.450 | 0.251 | 0.607 | 0.207 |
| **SCGF_beta** | baseline model + inflammatory exposures | 0.163 | -0.145 | 0.472 | 0.294 | 0.606 | 0.208 |
| **SCGF_beta** | adjusted model | 0.117 | -0.152 | 0.386 | 0.388 | 0.873 | 0.335 |
| **SCGF_beta** | adjusted model + inflammatory exposures | 0.093 | -0.202 | 0.388 | 0.531 | 0.866 | 0.330 |
| **Semaphorin.3E** | univariable model | 0.079 | -0.168 | 0.325 | 0.528 | 0.645 | -0.009 |
| **Semaphorin.3E** | baseline model | 0.134 | -0.086 | 0.355 | 0.228 | 0.607 | 0.209 |
| **Semaphorin.3E** | baseline model + inflammatory exposures | 0.138 | -0.089 | 0.366 | 0.229 | 0.606 | 0.212 |
| **Semaphorin.3E** | adjusted model | 0.086 | -0.124 | 0.296 | 0.417 | 0.873 | 0.334 |
| **Semaphorin.3E** | adjusted model + inflammatory exposures | 0.088 | -0.128 | 0.305 | 0.417 | 0.850 | 0.333 |
| **SKR3** | univariable model | 0.112 | -0.134 | 0.359 | 0.365 | 0.574 | -0.003 |
| **SKR3** | baseline model | -0.077 | -0.333 | 0.179 | 0.550 | 0.754 | 0.195 |
| **SKR3** | baseline model + inflammatory exposures | -0.099 | -0.366 | 0.168 | 0.462 | 0.651 | 0.200 |
| **SKR3** | adjusted model | -0.018 | -0.260 | 0.224 | 0.881 | 0.929 | 0.327 |
| **SKR3** | adjusted model + inflammatory exposures | -0.022 | -0.277 | 0.233 | 0.865 | 0.931 | 0.325 |
| **SIGLEC1** | univariable model | -0.250 | -0.490 | -0.010 | 0.042 | 0.191 | 0.048 |
| **SIGLEC1** | baseline model | -0.113 | -0.397 | 0.170 | 0.427 | 0.696 | 0.199 |
| **SIGLEC1** | baseline model + inflammatory exposures | -0.131 | -0.421 | 0.159 | 0.370 | 0.606 | 0.203 |
| **SIGLEC1** | adjusted model | -0.043 | -0.315 | 0.229 | 0.754 | 0.929 | 0.328 |
| **SIGLEC1** | adjusted model + inflammatory exposures | -0.037 | -0.323 | 0.250 | 0.799 | 0.931 | 0.326 |
| **SLITRK5** | univariable model | 0.239 | -0.001 | 0.480 | 0.051 | 0.191 | 0.043 |
| **SLITRK5** | baseline model | 0.000 | -0.255 | 0.255 | 1.000 | 1.000 | 0.190 |
| **SLITRK5** | baseline model + inflammatory exposures | 0.044 | -0.223 | 0.312 | 0.742 | 0.907 | 0.194 |
| **SLITRK5** | adjusted model | 0.049 | -0.194 | 0.292 | 0.689 | 0.929 | 0.328 |
| **SLITRK5** | adjusted model + inflammatory exposures | 0.067 | -0.188 | 0.322 | 0.599 | 0.931 | 0.329 |
| **Thrombopoietin_receptor** | univariable model | 0.315 | 0.080 | 0.550 | 0.009 | 0.082 | 0.086 |
| **Thrombopoietin_receptor** | baseline model | 0.118 | -0.204 | 0.441 | 0.466 | 0.707 | 0.197 |
| **Thrombopoietin_receptor** | baseline model + inflammatory exposures | 0.076 | -0.266 | 0.418 | 0.658 | 0.852 | 0.195 |
| **Thrombopoietin_receptor** | adjusted model | 0.086 | -0.228 | 0.400 | 0.584 | 0.929 | 0.330 |
| **Thrombopoietin_receptor** | adjusted model + inflammatory exposures | 0.072 | -0.257 | 0.400 | 0.663 | 0.931 | 0.327 |
| **Trypsin.2** | univariable model | -0.214 | -0.456 | 0.028 | 0.082 | 0.259 | 0.031 |
| **Trypsin.2** | baseline model | -0.165 | -0.392 | 0.063 | 0.153 | 0.561 | 0.217 |
| **Trypsin.2** | baseline model + inflammatory exposures | -0.159 | -0.396 | 0.078 | 0.184 | 0.606 | 0.217 |
| **Trypsin.2** | adjusted model | -0.130 | -0.355 | 0.094 | 0.250 | 0.873 | 0.342 |
| **Trypsin.2** | adjusted model + inflammatory exposures | -0.170 | -0.405 | 0.066 | 0.154 | 0.850 | 0.351 |
| **VCAM1** | univariable model | -0.063 | -0.310 | 0.185 | 0.614 | 0.711 | -0.011 |
| **VCAM1** | baseline model | 0.048 | -0.192 | 0.289 | 0.690 | 0.893 | 0.192 |
| **VCAM1** | baseline model + inflammatory exposures | 0.016 | -0.236 | 0.267 | 0.901 | 0.922 | 0.192 |
| **VCAM1** | adjusted model | 0.052 | -0.175 | 0.279 | 0.647 | 0.929 | 0.329 |
| **VCAM1** | adjusted model + inflammatory exposures | 0.030 | -0.210 | 0.269 | 0.804 | 0.931 | 0.326 |
| **VEGFA** | univariable model | -0.199 | -0.442 | 0.044 | 0.106 | 0.292 | 0.025 |
| **VEGFA** | baseline model | -0.014 | -0.261 | 0.234 | 0.913 | 1.000 | 0.190 |
| **VEGFA** | baseline model + inflammatory exposures | -0.033 | -0.286 | 0.221 | 0.797 | 0.914 | 0.193 |
| **VEGFA** | adjusted model | -0.013 | -0.242 | 0.215 | 0.908 | 0.929 | 0.327 |
| **VEGFA** | adjusted model + inflammatory exposures | -0.028 | -0.265 | 0.208 | 0.810 | 0.931 | 0.326 |
| **WFIKKN2** | univariable model | 0.266 | 0.028 | 0.505 | 0.029 | 0.184 | 0.057 |
| **WFIKKN2** | baseline model | 0.141 | -0.099 | 0.381 | 0.245 | 0.607 | 0.208 |
| **WFIKKN2** | baseline model + inflammatory exposures | 0.131 | -0.124 | 0.386 | 0.307 | 0.606 | 0.207 |
| **WFIKKN2** | adjusted model | 0.108 | -0.116 | 0.332 | 0.338 | 0.873 | 0.337 |
| **WFIKKN2** | adjusted model + inflammatory exposures | 0.088 | -0.149 | 0.324 | 0.461 | 0.850 | 0.332 |
|  |  |  |  |  |  |  |  |

| **group1** | **group2** | **n1** | **n2** | **statistic** | **p** | **pFDR (q)** |
| --- | --- | --- | --- | --- | --- | --- |
| 0 inflammatory exposures | 1 inflammatory exposure | 104 | 67 | 2575 | 0.004 | 0.012 |
| 0 inflammatory exposures | 2 inflammatory exposures | 104 | 42 | 1549 | 0.006 | 0.012 |
| 0 inflammatory exposures | 3+ inflammatory exposures | 104 | 18 | 417 | <0.001 | 0.001 |
| 1 inflammatory exposure | 2 inflammatory exposures | 67 | 42 | 1407 | 1 | 1 |
| 1 inflammatory exposure | 3+ inflammatory exposures | 67 | 18 | 429 | 0.062 | 0.074 |
| 2 inflammatory exposures | 3+ inflammatory exposures | 42 | 18 | 260 | 0.058 | 0.074 |

**Supplementary Table 12. Associations between DNAm CCL18 and number of inflammatory morbidities.** Post-hoc pairwise testing (Wilcoxon rank-sum tests) of inflammatory exposure categories.

| **Exposure** | **OR** | **Lower CI** | **Upper CI** | **p** |
| --- | --- | --- | --- | --- |
| NEC | 1.480 | 0.860 | 2.505 | 0.144 |
| BPD | 1.612 | 1.196 | 2.211 | 0.002 |
| sepsis | 1.790 | 1.298 | 2.526 | 0.001 |
| HCA | 1.284 | 0.958 | 1.727 | 0.095 |

**Supplementary Table 13. Associations between DNAm CCL18 and individual perinatal inflammatory morbidities.** Results are presented as odds ratios and 95% confidence intervals. Models are adjusted for gestational age at sampling, birthweight Z score and infant sex.

| **group1** | **group2** | **n1** | **n2** | **statistic** | **p** | **pFDR (q)** |
| --- | --- | --- | --- | --- | --- | --- |
| 0 inflammatory exposures | 1 inflammatory exposure | 104 | 67 | 2804 | 0.032 | 0.189 |
| 0 inflammatory exposures | 2 inflammatory exposures | 104 | 42 | 1853 | 0.153 | 0.459 |
| 0 inflammatory exposures | 3+ inflammatory exposures | 104 | 18 | 835 | 0.468 | 0.936 |
| 1 inflammatory exposure | 2 inflammatory exposures | 67 | 42 | 1429 | 0.894 | 0.987 |
| 1 inflammatory exposure | 3+ inflammatory exposures | 67 | 18 | 605 | 0.987 | 0.987 |
| 2 inflammatory exposures | 3+ inflammatory exposures | 42 | 18 | 382 | 0.955 | 0.987 |

**Supplementary Table 14. Associations between DNAm CRTAM and number of inflammatory morbidities.** Post-hoc pairwise testing (Wilcoxon rank-sum tests) of inflammatory exposure categories.

| **Exposure** | **OR** | **Lower CI** | **Upper CI** | **p** |
| --- | --- | --- | --- | --- |
| NEC | 1.055 | 0.558 | 1.918 | 0.865 |
| BPD | 1.414 | 1.053 | 1.919 | 0.023 |
| sepsis | 0.905 | 0.656 | 1.237 | 0.536 |
| HCA | 1.186 | 0.890 | 1.584 | 0.243 |

**Supplementary Table 15. Associations between DNAm CRTAM and individual perinatal inflammatory morbidities.** Results are presented as odds ratios and 95% confidence intervals. Models are adjusted for gestational age at sampling, birthweight Z score and infant sex.
